# Genetic Dissection of Differential Signaling Threshold Requirements for the Wnt/β-Catenin Pathway *In Vivo*

**DOI:** 10.1371/journal.pgen.1000816

**Published:** 2010-01-15

**Authors:** Michael Buchert, Dimitris Athineos, Helen E. Abud, Zoe D. Burke, Maree C. Faux, Michael S. Samuel, Andrew G. Jarnicki, Catherine E. Winbanks, Ian P. Newton, Valerie S. Meniel, Hiromu Suzuki, Steven A. Stacker, Inke S. Näthke, David Tosh, Joerg Huelsken, Alan R. Clarke, Joan K. Heath, Owen J. Sansom, Matthias Ernst

**Affiliations:** 1 Ludwig Institute for Cancer Research, Royal Melbourne Hospital, Parkville, Australia; 2 The Beatson Institute Cancer Research, Garscube Estate, Glasgow, United Kingdom; 3 Department of Anatomy and Cell Biology, University of Melbourne, Melbourne, Australia; 4 Department of Anatomy and Developmental Biology, Monash University, Clayton, Australia; 5 Centre for Regenerative Medicine, Department of Biology and Biochemistry, University of Bath, Bath, United Kingdom; 6 Department of Surgery, University of Melbourne, Parkville, Australia; 7 Cell and Developmental Biology, University of Dundee, Dundee, United Kingdom; 8 School of Biosciences, University of Cardiff, Cardiff, United Kingdom; 9 First Department of Internal Medicine, Sapporo Medical University, Sapporo, Japan; 10 Ecole Polytechnique Fédérale de Lausanne, Swiss Institute for Experimental Cancer Research, Lausanne, Switzerland; Stanford University School of Medicine, Howard Hughes Medical Institute, United States of America

## Abstract

Contributions of null and hypomorphic alleles of *Apc* in mice produce both developmental and pathophysiological phenotypes. To ascribe the resulting genotype-to-phenotype relationship unambiguously to the Wnt/β-catenin pathway, we challenged the allele combinations by genetically restricting intracellular β-catenin expression in the corresponding compound mutant mice. Subsequent evaluation of the extent of resulting Tcf4-reporter activity in mouse embryo fibroblasts enabled genetic measurement of Wnt/β-catenin signaling in the form of an allelic series of mouse mutants. Different permissive Wnt signaling thresholds appear to be required for the embryonic development of head structures, adult intestinal polyposis, hepatocellular carcinomas, liver zonation, and the development of natural killer cells. Furthermore, we identify a homozygous *Apc* allele combination with Wnt/β-catenin signaling capacity similar to that in the germline of the *Apc^min^* mice, where somatic *Apc* loss-of-heterozygosity triggers intestinal polyposis, to distinguish whether co-morbidities in *Apc^min^* mice arise independently of intestinal tumorigenesis. Together, the present genotype–phenotype analysis suggests tissue-specific response levels for the Wnt/β-catenin pathway that regulate both physiological and pathophysiological conditions.

## Author Summary

Germline or somatic mutations in genes are the underlying cause of many human diseases, most notably cancer. Interestingly though, even in situations where every cell of every tissue of an organism carries the same mutation (as is the case for germline mutations), some tissues are more susceptible to the development of disease over time than others. For example, in familial adenomatous polyposis (FAP), affected persons carry different germline mutations in the *APC* gene and are prone to developing cancers of the colon and the rectum—and, less frequently, cancers in other tissues such as stomach, liver, and bones. Here we utilize a panel of mutant mice with truncating or hypomorphic mutations in the *Apc* gene, resulting in different levels of activation of the Wnt/β-catenin pathway. Our results reveal that different pathophysiological outcomes depend on different permissive signaling thresholds in embryonic, intestinal, and liver tissues. Importantly, we demonstrate that reducing Wnt pathway activation by 50% is enough to prevent the manifestation of embryonic abnormalities and disease in the adult mouse. This raises the possibility of developing therapeutic strategies that modulate the activation levels of this pathway rather than trying to “repair” the mutation in the gene itself.

## Introduction

The evolutionarily conserved Wnt/β-catenin pathway is a critical regulator of proliferation and differentiation and plays a pivotal role during embryonic development and in the maintenance of tissue homeostasis in the adult. A multitude of studies have documented that impaired or excessive activation of the Wnt/β-catenin pathway result in a large number of pathophysiological conditions, including cancer (for review see [1]). Tight regulation of Wnt/β-catenin signaling is ensured by compartmentalized expression of the different Wnt ligands and receptor components and this is complemented by multiple layers of negative regulation. In particular, the tumor suppressor protein Apc provides a platform for the formation of a β-catenin destruction complex, and thereby acts as a negative regulator of activated Wnt signaling. Loss of Apc function leads to ligand-independent accumulation of β-catenin and its nuclear translocation, where it binds to Tcf/Lef family transcription factors and induces expression of target genes such as *Axin2*, *Cyclin D1* and *c-Myc* that are involved in proliferation and transformation (for review see [2]).

During embryonic development, Wnt/β-catenin signaling plays an important role in the anterior-posterior patterning of the primary embryonic axis in vertebrates. Unregulated activity of the Wnt pathway during embryonic development leads to anterior defects. For example in mice, loss of *Dkk1,* a Wnt antagonist, results in truncation of head structures anterior to the mid-hindbrain boundary [3] and mice doubly deficient for the Wnt antagonists *Sfrp1* and *Sfrp2* have a shortened anterior-posterior axis [4]. Ectopic expression of Wnt8C in mice causes axis duplication and severe anterior truncations [5], while embryos lacking functional β-catenin have impaired anterior-posterior axis formation [6]. Embryos homozygous for the mutant *Apc^min^* allele, which results in truncation of the full-length 2843 amino acid protein at residue 850 and in heterozygous mice leads to an intestinal phenotype akin to familial adenomatous polyposis (FAP) in humans, fail to develop past the gastrulation stage due to proximalisation of the epiblast and ectopic activation of several posterior mesendodermal genes [7],[8]. While these observations establish indispensable roles for components of the Wnt pathway in patterning the anterior-posterior axis, recent genetic rescue studies have helped to define signaling threshold requirement(s) for head morphogenesis [9].

Mutations in components of the destruction complex (APC, AXIN, GSK3β etc) are implicated in tumorigenesis and result in aberrant, ligand-independent activation of the WNT/β-CATENIN pathway. For instance truncating nonsense mutations in *APC*, loss of heterozygosity (LOH) or promoter hypermethylation are most prominently associated with aberrant WNT signaling that is characteristic of more than 90% of sporadic forms of colorectal cancer in humans [10]–[12]. Meanwhile epigenetic and genetic impairment mutations that reduce expression of wild-type AXIN2/CONDUCTIN [13],[14] or amino-terminal missense mutations in CTNNB1 (β-CATENIN) [15] are most commonly associated with aberrant WNT signaling in cancers of the liver (hepatocellular carcinoma and hepatoblastoma), stomach, kidney (Wilms tumor) and ovaries. It remains unclear why in humans the intestinal epithelium is most sensitive to cancer-associated somatic mutations in APC rather than to those in other components of the WNT signaling cascade, and to what extent this may be due to the loss of interaction between APC and actin-regulatory proteins and microtubules that affect cell migration, orientation, polarity, division and apoptosis, rather than the proliferation/differentiation generally associated with WNT/β-CATENIN signaling (for review see [16]). However, at least in the mouse the C–terminal domains of Apc are dispensable for its tumor suppressing functions [17]. In addition, phenotypic changes observed after the conditional deletion of Apc including those on apoptosis, migration, differentiation and proliferation are rescued by concomitant deletion of the Wnt/β-catenin target gene *Myc* [18].

Signaling threshold levels *in vivo* have been assessed by various approaches, including administration of (ant-) agonistic compounds, the (inducible) over-expression of transgenes and the creation of haploinsufficiency through the combination of knock-out and hypomorphic alleles. Elegant combinations of different hypomorphic *Apc* alleles, for instance, have demonstrated that within the context of intestinal tumorigenesis, there is a clear correlation between gene dosage and phenotype severity [17],[19]. In particular, these studies implied an inverse correlation between the level of Apc protein expression and activation of the Wnt/β-catenin pathway, and in turn, proliferation and differentiation of epithelium along the crypt-villus axis as well as cell renewal in the stem cell compartment [20]. Here we genetically identify differences in signaling threshold levels that determine physiological and pathological outcomes during embryonic development and various aspects of tissue homeostasis in adult tissue. Using combinations of epistatically related hypomorphic alleles of components of the Wnt/β-catenin signaling cascade, we identify tissue-specific signaling threshold levels for anterior specification during embryogenesis, intestinal and hepatic homeostasis in the adult. Our observations add further support to the “just-right” model [21] of Wnt/β-catenin signaling activation where distinct dosages are required to perturb the self-renewal of stem cell populations and lead to neoplastic transformation in the intestine and liver.

## Results/Discussion

### Genetic modulation of full-length Apc expression in mouse embryonic fibroblasts

**Figure 1:**
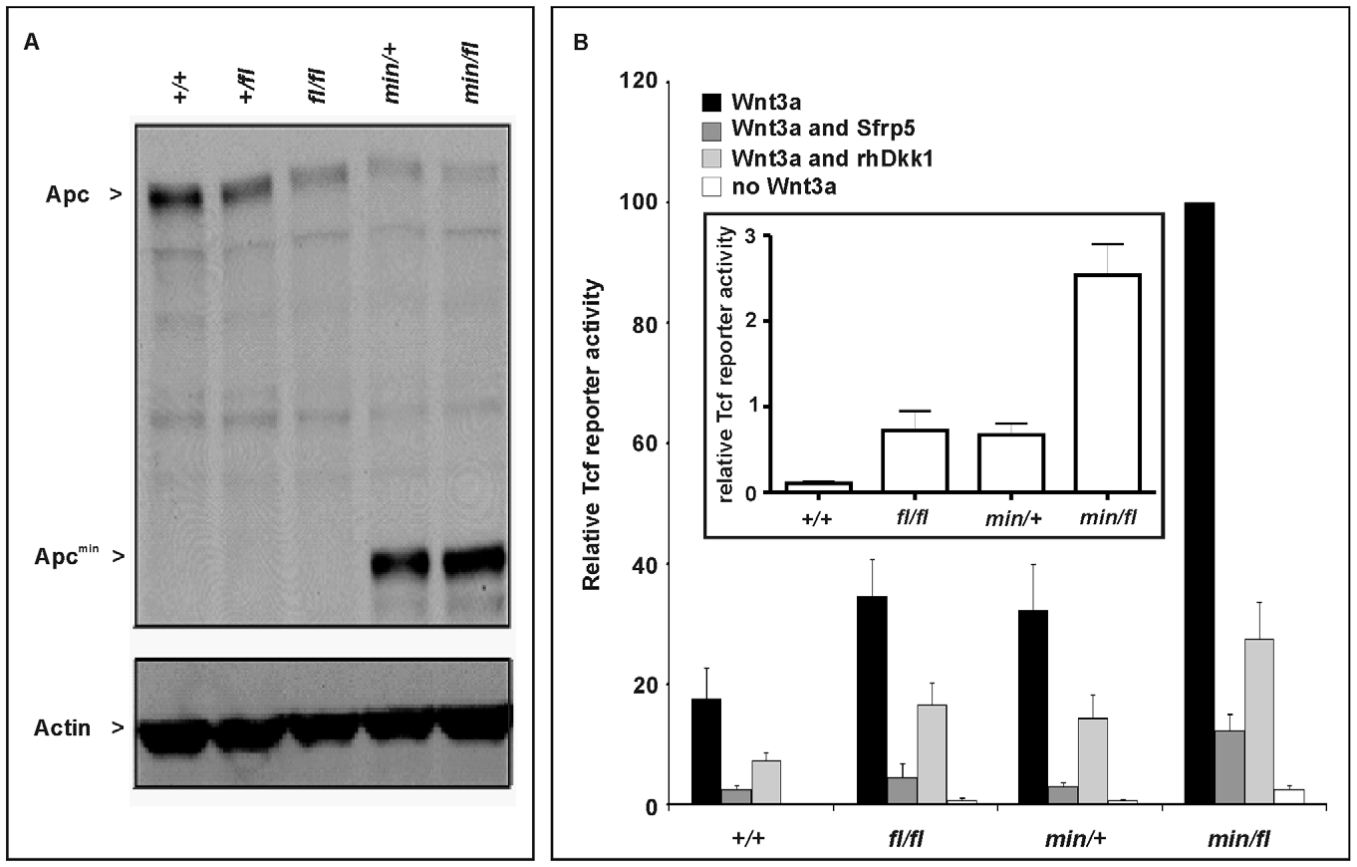
Apc protein expression levels and Wnt/β-catenin pathway activation in primary mouse embryonic fibroblasts (MEFs). (A) Immunoblot analysis of MEF lysates prepared from mice of the indicated genotype were used to detect full-length (Apc) and mutant (Apc^min^) protein using β-actin as loading control. (B) Tcf4 reporter activity in response to Wnt3a in the presence or absence of the antagonists Dkk1 or Sfrp5. Activity was assessed following transient transfection of the p*SuperTopFlash* reporter plasmid. Where indicated, cells were cotransfected with an expression plasmid encoding Sfrp5, and stimulated with recombinant human Dkk1 and conditioned medium from cells expressing Wnt3a. Cultures were harvested 48h later and assayed for luciferase activity using the dual luciferase system and reporter activity in (*min/fl*) MEF exposed to a submaximal Wnt3a stimulation was arbitrarily set to 100. At least two independent experiments were performed in triplicates for each genotype. The insert shows relative Tcf4 reporter activation in the absence of stimulation with Wnt3a ligand. Mean ± SD. Genotypes are as follows: wild-type (*+/+*); *Apc^+/fl^* (*+/fl*); *Apc^fl/fl^* (*fl/fl*); *Apc^min/+^* (*min/+*); *Apc^min/fl^* (*min/fl*). All MEFs were derived from mice on a mixed genetic 129Sv x C57BL/6 background.

In order to modulate the activity of the Wnt/β-catenin pathway in the mouse, we took advantage of the *Apc^min^* [22] and *Apc^fl^* [23] alleles. The premature stop codon encoded by the *Apc^min^* allele encodes a truncated 850 amino acid Apc protein, which lacks the 15- and 20 aa repeats and Axin binding repeats required for β-catenin regulation [24], while the unrecombined *Apc^fl^* allele results in attenuated expression levels of wild-type *Apc* mRNA [23]. We used Western blot analysis of lysates from mouse embryo fibroblasts (MEFs) to quantitate expression of full-length Apc protein and the capacity to augment Wnt3a-dependent signaling in cells from the corresponding *Apc* allele combinations. We observed an inverse relationship in the hierarchy of allele combinations between full-length Apc protein expression (Figure 1A), and signaling activity of the Wnt/β-catenin pathway recorded with a Tcf4 reporter plasmid (Figure 1B). Owing to the presence of residual amounts of full-length Apc protein, the two soluble Wnt antagonists Sfrp5 and Dkk1 were able to suppress Wnt3a-mediated reporter activation in cells of all tested allele combinations. However, in the presence of Wnt3a, p*SUPERTopFlash* reporter activity was inhibited less effectively by Sfrp5 and Dkk1 in cells with impaired expression of full-length Apc protein (Figure 1B). Therefore, genetic modulation of the expression levels of full-length Apc protein enables experimental manipulation of Wnt/β-catenin pathway activation for a given concentration of Wnt ligand or its soluble antagonists.

**Figure 2:**
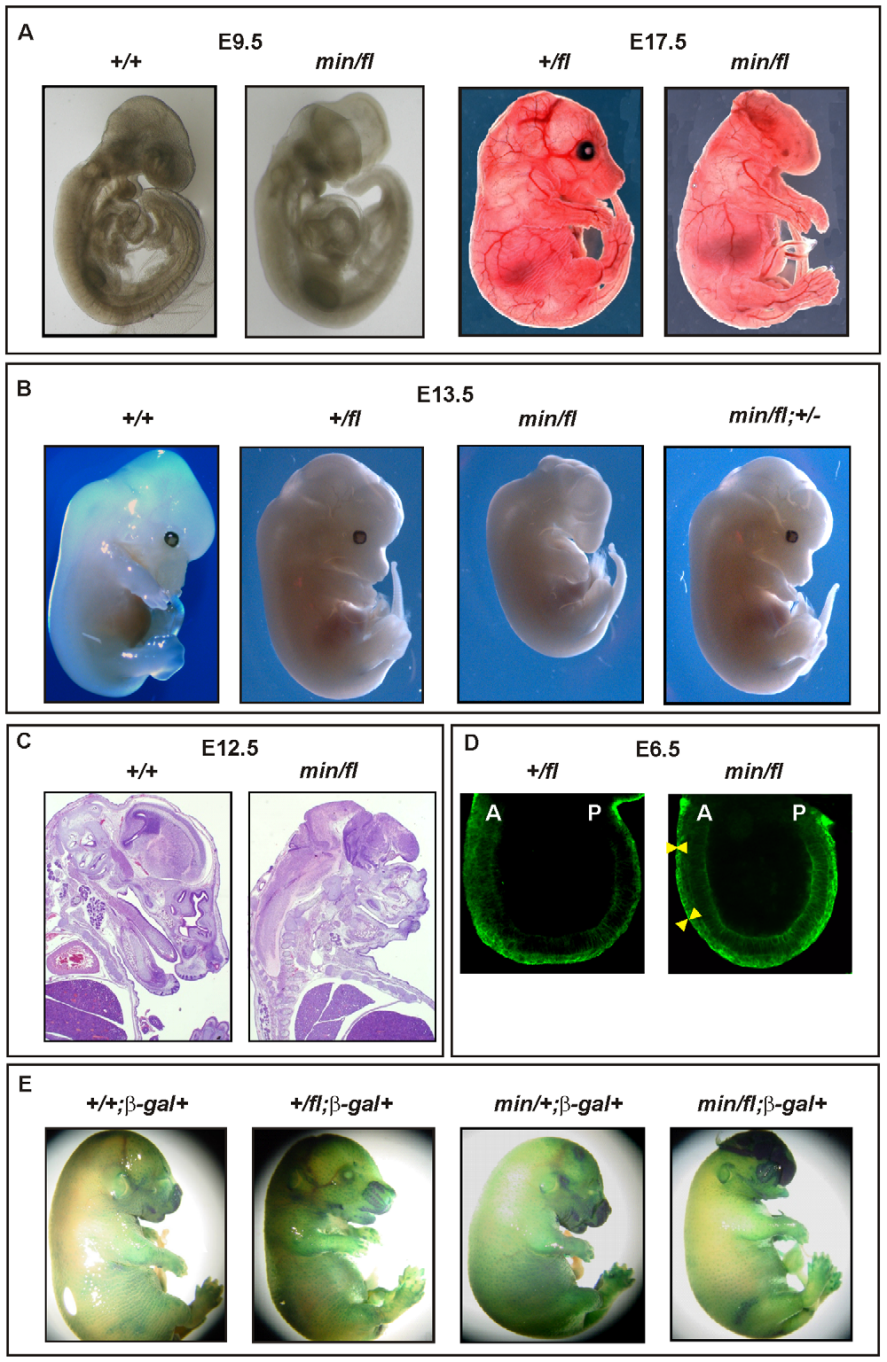
Excessive Wnt/β-catenin signaling results in anterior head defects during embryonic development. (A) Whole mounts of E9.5 (*+/+*) and (*min/fl*) mutant embryos and E17.5 (*+/fl*) and (*min/fl*) mutant embryos. (B) Whole mounts of E13.5 wild-type and mutant embryos of the indicated genotypes. Genetic ablation of one allele of β-catenin in (*min/fl*) “headless” mutant rescues normal head morphology in (*min/fl;* +/−) mice. (C) Histological cross sections of E12 wild-type (*+/+*) and mutant (*min/fl*) embryos. (D) Confocal cross section of E3.5–5.5 (*+/fl*) and (*min/fl*) embryos stained for β-catenin protein. The arrowheads demarcate the outer layer (anterior visceral endoderm) of the embryo which shows increased and expanded β-catenin expression in the (*min/fl*) mutant. A, Anterior; P, Posterior. (E) Whole mount *in vivo* X-gal staining of E16 embryos to monitor canonical Wnt/β-catenin dependent activity in compound mutant mice harboring the corresponding *BAT*::gal reporter transgene. Genotypes are as follows: wild-type (*+/+*); *Apc^+/fl^* (*+/fl*); *Apc^fl/fl^* (*fl/fl*); *Apc^min/+^* (*min/+*); *Apc^min/fl^* (*min/fl*); *Apc^min/fl^;Ctnnb1^+/−^* (*min/fl;* +/−). All mice were on a mixed genetic 129Sv x C57BL/6 background.

To assess whether the outcome of incremental modulation of Wnt/β-catenin signaling by genetic means in MEFs would impact differentially during development and in adult tissue homeostasis *in vivo*, we set out to generate adult mutant mice with genotypes comprising different combinations of *Apc* alleles. Surprisingly, we were unable to obtain *Apc^min/fl^* mice at term from crossing heterozygous *Apc^+/fl^* with *Apc^min/+^* mice. Since homozygous *Apc^min^*, but not *Apc^fl^*, mice die *in utero* due to gastrulation defects [7], we genotyped 117 embryos at E12 and found that all 30 *Apc^min/fl^* embryos lacked all structures anterior to the hindbrain. Anterior morphological defects first became visible in E8.5-E9.5 *Apc^min/fl^* embryos, and remained restricted to that region throughout embryonic development (Figure 2A and 2B). Histological cross-sections of *Apc^min/fl^* E12 embryos revealed the presence of a prominent cap of neural tissue that formed at the most anterior part of the embryo, in the absence of cranial structures and the mandible (Figure 2C). Next we used the *BAT::*gal reporter allele to confirm excessive Tcf4-dependent β-galactosidase reporter activity in the neural tissue cap of *Apc^min/fl^* E15 embryos. As predicted from the Tcf-reporter analysis in MEFs, we also observed *BAT*::gal reporter activity around the fronto-nasal region with a gradual increase from *Apc^+/+^* to *Apc^+/fl^* and *Apc^min/+^* embryos. This was further extended to most abnormal anterior structures in the *Apc^min/fl^* embryos (Figure 2E). Furthermore, analysis of E5.5-E7.5 embryos by wholemount confocal immunohistochemistry revealed anterior extension of β-catenin expression in the anterior visceral endoderm, an axial signaling centre in the outer endoderm layer of early embryos [8],[25], of *Apc^min/fl^* embryos when compared to their *Apc^+/fl^* counterparts (Figure 2D). However, “headless” *Apc^min/fl^* embryos were present at the expected Mendelian ratios until E15.5 (Table S1A) and live embryos could still be detected at E17.5 (Theiler stage 25–26) (Figure 2A) but at less than the expected Mendelian ratio. Our observations therefore support a role for limiting Apc-dependent signaling) functions during the development and patterning of the most anterior structures of the embryo similar to that proposed for excessive Wnt3 signaling in *Dkk1*-deficient or compound mutant *Dkk1^+/−^;Wnt3^+/−^* mice [9],[20], and reminiscent of the function played by *Otx2* [26].

To establish that the “headless” phenotype in *Apc^min/fl^* mice arose from altering the extent of Wnt/β-catenin signaling rather than arising from other potentially dominant-negative activities mediated by the truncated *Apc^min^* protein, we conducted three further genetic experiments. First, we created a more severely 580 amino acid truncated Apc protein by excising exon 14 in *Apc^+/fl^* mice that were crossed with the *CMV*:Cre deletor strain to induce a germline nonsense frame-shift mutation in the corresponding recombined *Apc^580Δ^* allele. Subsequent matings of Cre-transgene-negative *Apc^580Δ/+^* mice with *Apc^+/fl^* mice failed to yield *Apc^580Δ/fl^* pups at birth (Table S1B). Meanwhile, inspection of E9.5, E12 and E16 litters revealed that approximately 25% of all embryos displayed a “headless” phenotype indistinguishable from that observed in stage-matched *Apc^min/fl^* mice (data not shown).

Second, we attempted to rescue the “headless” phenotype in *Apc^min/fl^* mice by genetically limiting expression of β-catenin in corresponding *Apc^min/fl^;Ctnnb1^+/−^* compound mutant mice. Resulting *Apc^min/fl^;Ctnnb1^+/−^* MEFs revealed an approximately 50% reduction of Wnt/β-catenin signaling when compared to their *Apc^min/fl^;Ctnnb1^+/+^* counterparts (see below). When mating *Apc^fl/fl^;Ctnnb1^+/−^* with *Apc^min/+^;Ctnnb1^+/+^* mice, we recovered *Apc^+/fl^;Ctnnb1^+/+^, Apc^+/fl^;Ctnnb1^+/−^* and *Apc^min/fl^;Ctnnb1^+/−^* mice at weaning age at a similar ratio, while among E13.5 embryos, all four possible genotypes were represented at comparable frequencies (Table S1C and Figure S1). Importantly, *Apc^min/fl^;Ctnnb1^+/−^* mice developed normally into fecund adults (Figure 2B and data not shown), suggesting that limiting Wnt/β-catenin signaling corrected the development of detrimental phenotypes observed in *Apc^min/fl^* mice.

Since the atypical Wnt receptor component Ryk has recently been suggested to amplify Wnt signaling during cortical neurogenesis through β-catenin-dependent as well as independent pathways [27], we also tested whether the “headless” phenotype was promoted by Ryk activity. However, and in contrast to β-catenin, the embryonic lethality of *Apc^min/fl^* mice was not rescued by genetically limiting the expression of the atypical tyrosine kinase Ryk, because we failed to recover either *Apc^min/fl^;Ryk^+/−^* or *Apc^min/fl^;Ryk^-/-^* compound mutant mice at weaning (Table S1D), suggesting that Ryk expression was not contributing to the Wnt/β-catenin induced phenotype.

Collectively, our observations extend previous reports that identified a Wnt signaling gradient along the anterior-posterior axis and a requirement for Dkk1 and other Wnt antagonists at the anterior end to prevent posteriorization [3]–[6],[28],[29]. In particular, our experiments clarify genetically that the tight signaling requirements for head morphogenesis previously attributed to Apc or the extracellular components Dkk1 [3], Sfrp [4],[30], Wnt3a [9] and Wnt8a [5] occur exclusively through the Wnt/β-catenin pathway.

Unlike *Apc^min/fl^* embryos, *Apc^min/min^* embryos die around the time of gastrulation [7], consistent with our observation that *Apc^580Δ/min^* MEFs, which serve as a model for unavailable *Apc^min/min^* counterparts, reveal higher Tcf4 reporter activity than *Apc^min/fl^* MEFs (see below). Since the morphological defects in E4.75 *Apc^min/min^* embryos correlate with excessive nuclear β-catenin in the epiblast and primitive ectoderm [8], we also examined the effect of genetically limiting β-catenin in these embryos. Unlike the phenotypic rescue observed in *Apc^min/fl^;Ctnnb1^+/−^* mice, we detected *Apc^min/min^*;*Ctnnb1^+/−^* embryos only at E4.5 and E5.5 but not at later stages (E6 and E7). This finding is reminiscent of the time points of embryonic death of *Apc^min/min^* embryos [7] and suggested that reduction of Wnt/β-catenin signaling was insufficient to rescue their death immediately after gastrulation (data not shown). Therefore, higher threshold levels of Wnt/β-catenin signaling selectively inhibit development at an earlier stage (i.e. gastrulation) and genetic reduction of Wnt/β-catenin signaling through ablation of one *Ctnnb1* allele reduces signaling only below the threshold that is tolerated during later stages of development. However, we cannot formally exclude other essential function(s) of the full-length Apc protein, which could be provided by residual full-length protein encoded by the *Apc^fl^* allele, and which may be required around the time of gastrulation.

### Threshold levels in intestinal polyposis

**Figure 3:**
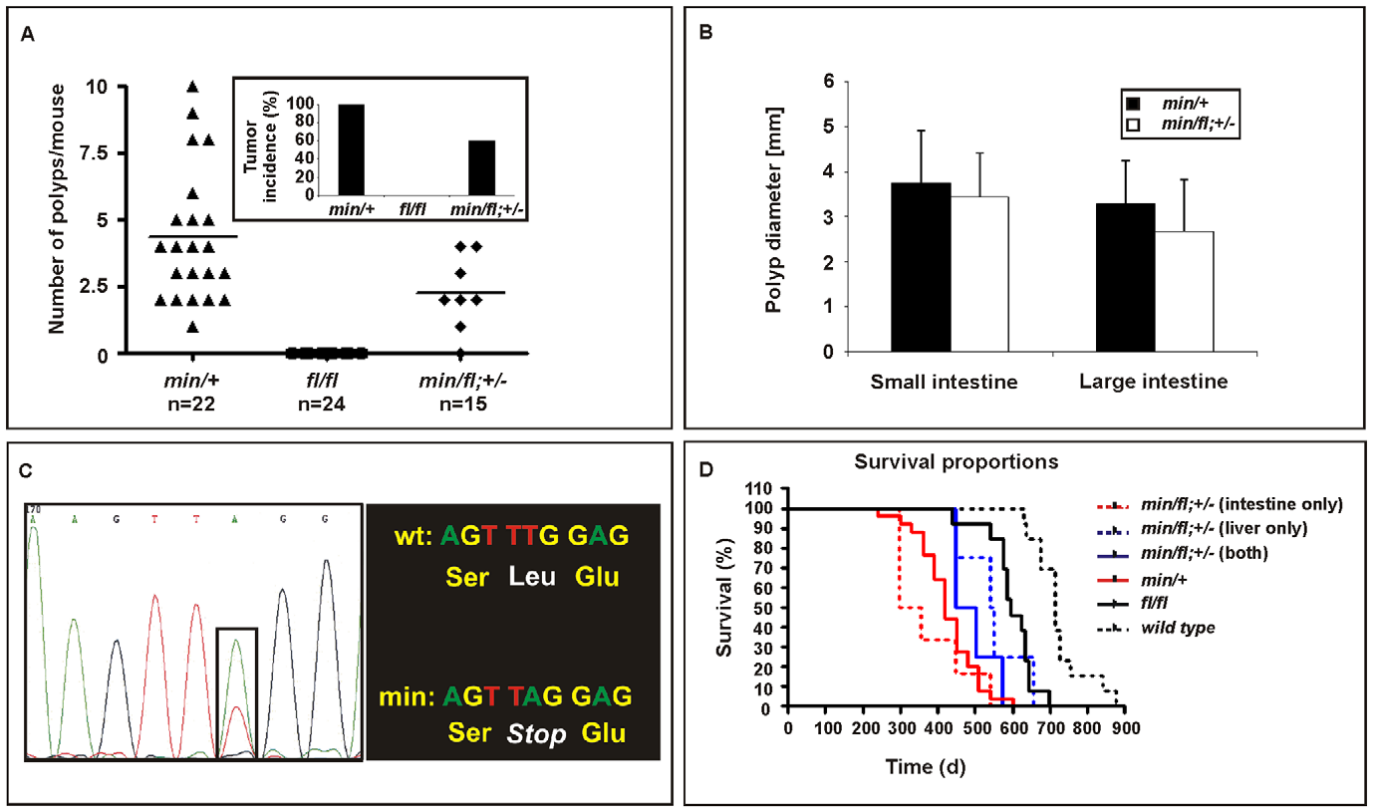
Intestinal tumor burden and impaired survival in *Apc* compound mutant mice. (A) Multiplicity of large (>2mm) tumors in individual moribund mice of the indicated genotypes. Insert shows overall tumor incidence in cohorts of mice of the indicated genotypes. (B) Multiplicity of large (>2 mm) tumors in the small and large intestine of individual moribund (*min/+*) and compound (*min/fl;* +/−) mice. (C) Representative allele-specific nucleotide sequence of DNA extracted from an intestinal tumor of a (*min/fl;* +/−) mouse. Samples were scored as having lost the wild-type allele when the ratio between the peak intensities (boxed area) was ≤0.6 [52]. (D) Survival curve of mice of the indicated genotypes. Livers and the intestines of (*min/fl;* +/−) mice were analyzed for macroscopic evidence of tumors before being allocated to cohorts with lesions confined to the indicated organ only. Genotypes are as follows: wild-type (*+/+*); *Apc^min/+^* (*min/+*); *Apc^fl/fl^* (*fl/fl*); *Apc^min/fl^;Ctnnb1^+/−^* (*min/fl;* +/−). All mice were on a mixed genetic 129Sv x C57BL/6 background.

*Apc^min/+^* mice develop intestinal polyposis upon spontaneous LOH of the wild-type *Apc* allele which arises from centromeric somatic recombination [31],[32]. Meanwhile, genetic studies estimated the polyposis threshold level to correspond to 10–15% of the full-length protein produced from biallelic *Apc* expression [19]. We therefore established aging cohorts of mice harbouring different *Apc* allele combinations to constitute an allelic series for Wnt/β-catenin signaling based on the results in Figure 1. As observed previously, *Apc^fl/fl^* mice on a mixed 129Sv x C57BL/6 background remained free of intestinal polyps (>18 month, n = 24), while all *Apc^min/+^* mice (n = 22) developed macroscopic lesions primarily within the proximal portion of the small intestine. Although tumor multiplicity and incidence was reduced in *Apc^min/fl^;Ctnnb1^+/−^* mice, leaving 6 of 15 mice (40%) free of polyps (Figure 3A), the remaining macroscopic lesions were of tubulo-villous structure and of similar size to those observed in age-matched *Apc^min^* mice (Figure 3B). The similar latency of disease onset between *Apc^min/+^* and *Apc^min/fl^;Ctnnb1^+/−^* mice suggests a common requirement for LOH. We therefore amplified exon 14 from polyps which contain the *min* allele-specific A>T transition to confirm LOH in all polyps from *Apc^min^* (n =  12) and *Apc^min/fl^;Ctnnb1^+/−^* mice (n =  4) (Figure 3C and data not shown). Based on our *in vitro* analysis (Figure 1), these results are similar to observations by Oshima *et al.* showing a requirement of less than 30% of wild-type Apc to prevent Wnt signaling from reaching the permissive threshold for intestinal polyps to form [33]. Surprisingly, restricting the pool of available cellular β-catenin in *Apc^min/fl^;Ctnnb1^+/−^* mice selectively reduced tumor multiplicity rather than tumor size when compared to *Apc^min^* mice. This suggests that, once LOH has occurred, Wnt/β-catenin signaling exceeds the permissive threshold level, even in light of a 50% reduction in β-catenin and fuels maximal tumor growth, which indeed may be mediated most effectively by submaximal Wnt activity [34].

### Threshold levels of hepatocellular carcinogenesis

**Figure 4:**
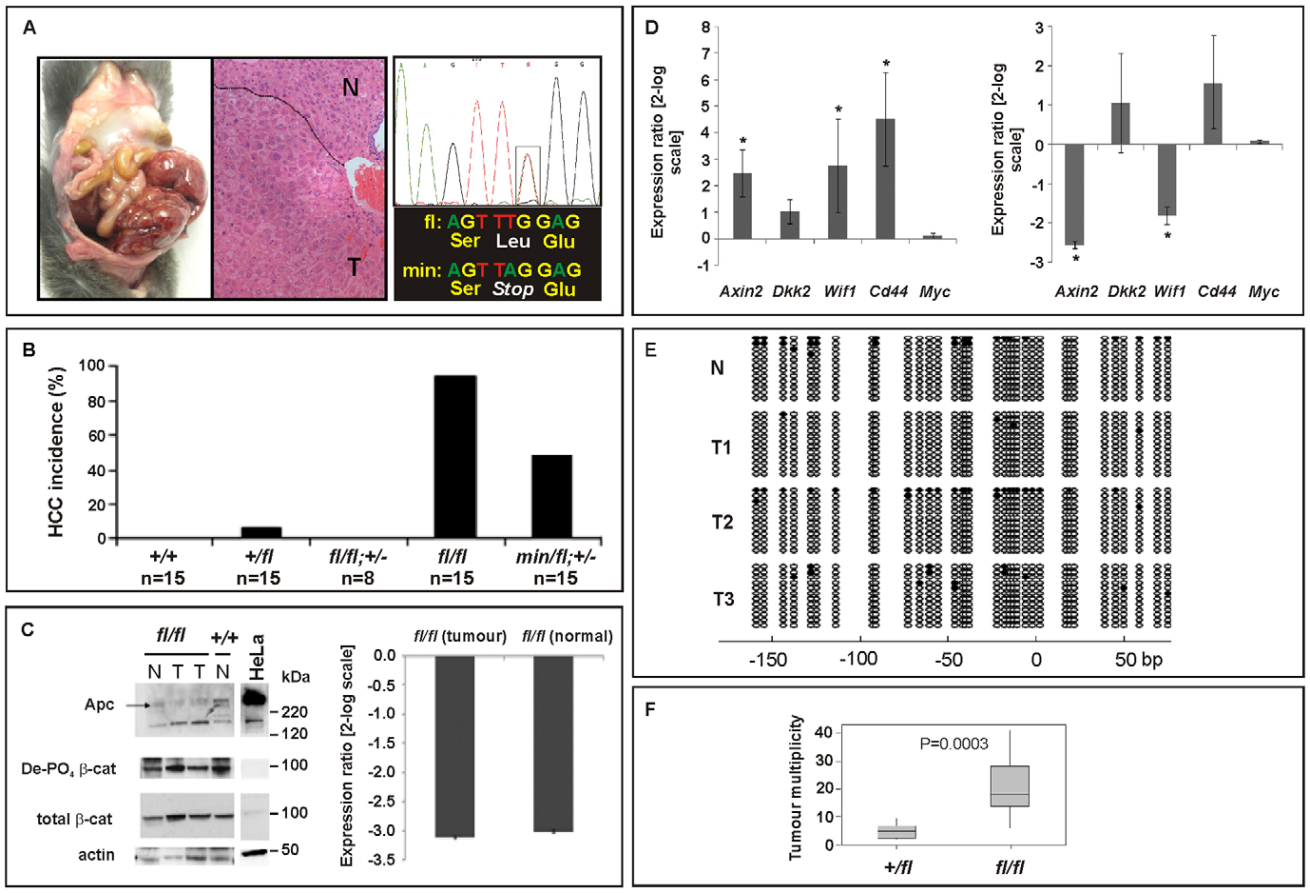
Liver phenotype in *Apc* mutant mice. (A) Hepatocellular carcinoma (HCC) of moribund *Apc^fl/fl^* and representative haematoxilin-eosin stained cross section with the dotted line indicating the boundary between normal (N) and tumoral (T) tissue. Representative allele-specific nucleotide sequence of DNA extracted from a liver tumor of a (*min/fl;* +/−) mouse demonstrating allelic balance between of the *Apc^min^* and the floxed wt allele (boxed area). (B) Incidence of HCC in mice of the indicated genotypes. (C) Western blot and qPCR analysis of full-length Apc protein and *Apc* mRNA in normal (N) and tumoral (T) liver tissue of *Apc^fl/fl^* and *Apc^+/+^* mice. Cell lysates of HeLa cells transfected with a plasmid encoding full-length wild-type Apc serves as an antibody specificity control. The abundance of de-phosphorylated, active (De-PO_4_) and total β-catenin protein in the same tissue extracts are shown with β-actin serving as a loading control. kDa, protein size marker in kilo Daltons. (D) Comparative qPCR analysis of representative Wnt target gene expression between normal and tumoral liver tissue collected from moribund *Apc^fl/fl^* mice (right panel). A comparable analysis was also performed on liver tissue from healthy 5mo old *Apc^fl/fl^* and wild-type mice (left panel). Mean ± SD with n≥3 mice per group. * P<0.05. (E) Bisulfite sequencing of the CpG island within the Axin2 promoter from adjacent normal (N) and tumor liver tissue (T1, T2, T3) from *Apc^fl/fl^* mice. Each vertical line refers to a CpG dinucleotide at the indicated position relative to the transcriptional start site. Following bisulfite-treatment, DNA was subcloned and sequenced. Horizontal lines represent individual sequences with open and full circles denoting unmethylated and methylated CpG residues, respectively. (F) Boxplot diagram comparing liver tumor multiplicity in *+/fl* mice (n = 9) and *fl/fl* mice (n = 12) 6 to 8 months after treatment with DEN. p = 0.0003 (Mann-Whitney). Genotypes are as follows: wild-type (*+/+*); *Apc^+/fl^* (*+/fl*); *Apc^fl/fl^* (*fl/fl*); *Apc^fl/fl^;Ctnnb1^+/−^* (*fl/fl;* +/−); *Apc^min/fl^;Ctnnb1^+/−^* (*min/fl;* +/−). All mice were on a mixed 129Sv x C57BL/6 background.

In humans, de-regulated WNT/β-catenin signaling plays an important role during onset and progression of hepatocellular carcinomas (HCC) and frequently arises from either dominant mutations in the *CTNNB1* (*β-catenin*) gene, or biallelic inactivation of the *AXIN1* and *AXIN2* genes that involves LOH associated with somatic (epi-)mutation [35]–[37]. Somatic *APC* mutations, by contrast, are rarely associated with liver carcinogenesis, but FAP patients with germline *APC* mutations frequently develop hepatoblastomas as well as colonic adenocarcinomas [38]. In addition, adenovirally transduced, complete *Apc* gene inactivation in the murine liver resulted in hepatomegaly-associated mortality [39], while its sporadic inactivation triggered the development of HCC [40]. We therefore assessed the incidence of liver tumors in moribund mice of the different *Apc* allele combinations. We found that all *Apc^fl/fl^* mice (n = 15), but none of their *Apc^fl/fl^;Ctnnb1^+/−^* littermates (n = 8), had developed HCC by 450 days of age (Figure 4A and 4B), but remained free of intestinal polyps (Figure 3A). We also used PCR analysis to exclude Cre-independent, spontaneous recombination of the *Apc^fl^* allele(s) in these tumors (Figure S2). Taken together with our observation of a reduced (but not complete loss) of Apc protein, this argues that tumors are formed with low level Apc and not in the absence of Apc. Therefore, our results suggest not only that HCC formation can occur due to excessive Wnt/β-catenin signaling but importantly that the permissive signaling threshold for hepatic tumorigenesis is lower than that for intestinal tumorigenesis consistently associated with LOH. Surprisingly, we observed HCC in 47% of *Apc^min/fl^;Ctnnb1^+/−^* mice (n = 15) including 20% that showed intestinal co-morbidity. Survival analysis of mice from this cohort, where disease was confined either to the intestine (n = 6) or the liver (n = 4; Figure 3D), suggested the requirement for a stochastic secondary event to occur akin to intestinal *Apc* LOH. However, our genomic analysis of hepatic biopsies from *Apc^min/fl^;Ctnnb1^+/−^* mice confirmed the absence of *Apc* LOH (Figure 4A), while qPCR and Western blot analysis revealed similar Apc expression between hepatic lesions and adjacent unaffected tissue from *Apc^fl/fl^* mice (Figure 4C). As expected, expression of Wnt target genes in unaffected livers from *Apc^fl/fl^* mice was elevated compared to livers from wt mice (Figure 4D). Meanwhile, in *Apc^fl/fl^* mice we found further, tumor-specific overexpression of some Wnt-target genes (incl. *Cd44*) that coincided with attenuation of others (notably encoding the negative regulators *Axin2, Dkk2* and *Wif1*).

In order to clarify the nature of potential additional somatic mutations that may affect or cooperate with Wnt/β-catenin signaling, we excluded the presence of activating mutations in *Ctnnb1*-exon3 that would ablate the negative regulatory phosphorylation sites in β-catenin (Table S2A). We also failed to identify aberrant hypermethylation of the proximal *Axin2* promoter (Figure 4E) and also excluded activating mutations in codons 12, 13 or 61 of *H-Ras* (Table S2B), although Harada et al. previously observed that simultaneous introduction of H-Ras and a constitutively active form of β-catenin by adenoviral gene transfer conferred HCC, while introduction of β-catenin alone did not [41],[42]. Indeed, exposure of *Apc^fl/fl^* mice to the liver-specific carcinogen diethylnitrosamine (DEN), which is known to promote mutations in *H-Ras*, resulted in a higher tumor incidence than in *Apc^+/fl^* mice (Figure 4F). Contrary to the observation with intestinal lesions collected from *Apc^min/fl^;Ctnnb1^+/−^* and *Apc^min^* mice, we found that hepatic tumor volumes in *Apc^fl/fl^* mice were larger than in *Apc^+/fl^* mice (261 mm^3^±167 mm^3^ (n = 12) *vs.* 193 mm^3^±414 mm^3^ (n = 9), p = 0.036; Mann-Whitney test; mean ± SEM; n = 9) suggesting that the extent of aberrant Wnt/β-catenin activity may control both initiation and progression of lesions in the liver.

**Figure 5:**
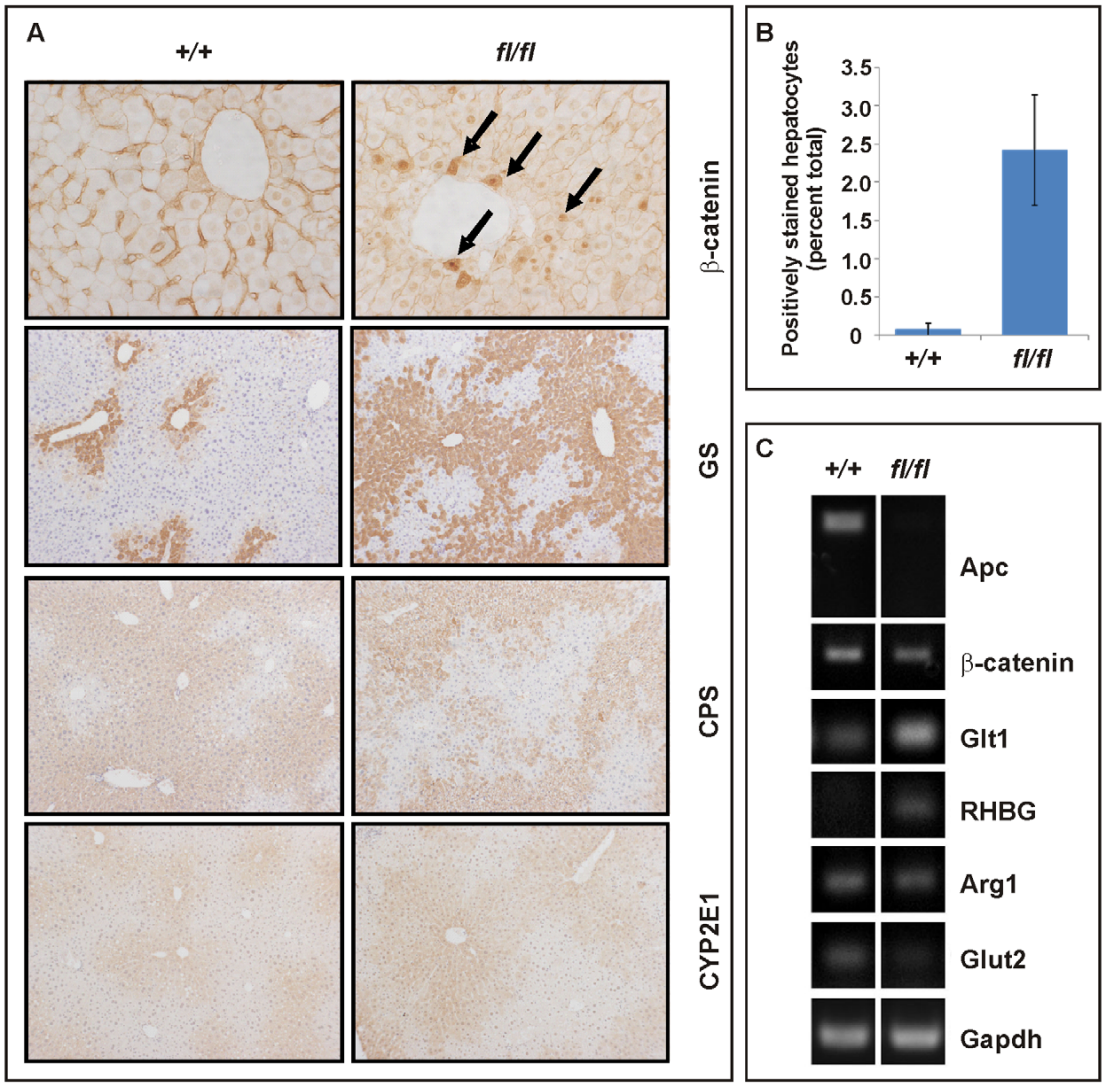
Liver zonation is affected in hypomorphic *Apc^fl/fl^* mutant mice. (A) Immunohistochemical expression analysis for β-catenin, glutamine synthetase (GS), carbamoylphosphate synthetase (CPS) and Cyp2E1 was performed on livers of age-matched wild-type (*+/+*) and (*fl/fl*) mice. Arrows point to nuclear β-catenin staining. (B) Hepatocytes with nuclear β-catenin staining were expressed as a percentage of total hepatocytes scored in age-matched wild-type (*+/+*; n = 3) and (*fl/fl*; n = 4) mice. p = 0.025 (Mann-Whitney). (C) Semi-quantitative RT-PCR analysis from liver of 5mo old healthy (*+/+*) and (*fl/fl*) mice for assessment of expression of *Apc*, *CtnnB1*, along with the perivenous markers *Glt1* (encoding a transporter of glutamate), *RHBG* (encoding the ammonium transporter), and the periportal markers *Arg1* (encoding arginase1) and *Glut2* (encoding glutaminase 2). *Gapdh* serves a as an RT-PCR amplification control.

Collectively, these data suggest differential signaling threshold requirements for intestinal and hepatic tumorigenesis and likely differences in the molecular mechanisms by which Wnt/β-catenin signaling promotes tumorigenesis in these two tissues. The relatively low proliferative activity of the hepatic stem cell compartment, for instance, may provide protection from *Apc* LOH, even when facilitated by haploinsufficient expression of a recQ-like DNA helicase in *Apc^min/+^;Blm^Cin/+^* compound mutant mice which remain free of HCC [43]. In light of the lack of *Axin2* promoter hypermethylation, the reduction of tumor-specific *Axin2* expression may arise from other stochastic events. For instance, the *AXIN2* locus contributes to some cancers by LOH or rearrangements in humans [44]. On the other hand, chronic inflammation and the associated excessive activation of the Interleukin-6 pathway may cooperate with activating mutations in *CTNNB1* during malignant transformation of human HCC [45]. Despite similar Tcf4 reporter activity recorded between *Apc^fl/fl^* and *Apc^min^* MEFs, *Apc^min^* mice remained free of HCC. This observation may be explained by the premature death of *Apc^min^* relative to *Apc^fl/fl^* mice (Figure 3D) together with the late onset of liver tumorigenesis. Indeed, we observe hepatic tumors in the *Apc^min/fl^;Ctnnb1^+/−^* mice which live longer than *Apc^min^* mice. On the other hand, hepatic tissue shows exquisite sensitivity to differential threshold levels of Wnt/β-catenin signaling, whereby the resulting signaling gradient provides a mechanism for metabolic liver zonation [39]. Indeed, we observed here that partial attenuation of full-length Apc expression in *Apc^fl/fl^* mice not only increased the number of cells with nuclear β-catenin (Figure 5A and 5B), but also altered expression of Wnt target genes and liver zonation. In particular, and in agreement with our previous findings [46], we observed that attenuation of full-length Apc favored expansion of a perivenous gene expression program (incl. *GS, Glt1* and *RHBG*) at the expense of a periportal signature (incl. *CPS*, *Arg1* and *Glut2*) (Figure 5A and 5C). Our observation that aberrant Wnt signaling in *Apc^fl/fl^* mice in the absence of additional somatic mutations in H-Ras bias towards tumors with perivenous characteristics is consistent with the finding that H-Ras mutated HCCs favor a periportal gene expression program [47].

### Reconciling tissue-specific phenotypes against different levels of Wnt/β-catenin signaling

**Figure 6:**
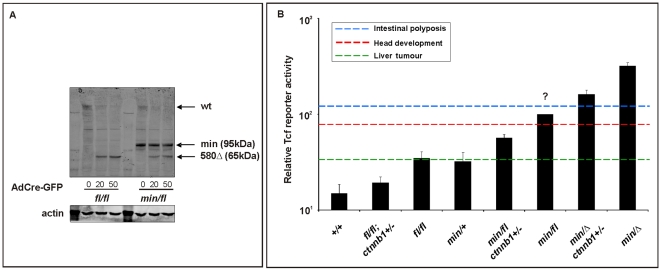
*In vitro* assessment of Wnt signaling in MEFs from *Apc* hypomorphic mice. (A) Western blot analysis of MEF cell lysates, prepared from (*fl/fl*) and (*min/fl*) mice, 48 h after infection with Cre-GFP expressing adenovirus (AdCre-GFP) at different concentrations. Note the gradual reduction of full-length Apc protein (wt) with increasing amount of AdCre-GFP administration and simultaneous accumulation of the truncated Apc^580Δ^ protein encoded by the recombined *Apc^fl^* allele. The truncated Apc protein encoded by the *Apc^min^* allele is indicated (min). (B) Tcf4 reporter activity in MEFs of the indicated genotype in response to a submaximally active concentration of Wnt3a-conditioned medium. Activity was assessed following transient transfection with p*SuperTopFlash*. Cells were harvested 48 h later and assayed for luciferase activity using the dual luciferase system. The activity of the Tcf4 reporter in (*min/fl*) MEF exposed to a submaximal Wnt3a stimulation was arbitrarily set to 100 and analysis was performed in triplicate cultures. Horizontal lines indicate the signaling threshold predicted for the indicated phenotypes to occur in mice of the corresponding genotypes. The question mark refers to the position of the “intestinal threshold” line relative to reporter activity in *min/fl* MEFs, since the corresponding adult min/fl mice can not be generated. At least two independent experiments were performed in triplicates for each genotype. Mean ± SD. Note: Histograms refer to situation before LOH, intestinal polyposis threshold to situation after LOH, where applicable. Genotypes are as follows: wild-type (*+/+*); *Apc^min/+^* (*min*/+); *Apc^fl/fl^* (*fl/fl*); *Apc^min/fl^* (*min/fl*); *Apc^min/Δ580^* (*min/Δ*); *Apc^fl/fl^;Ctnnb1^+/−^* (*fl/fl*;*ctnnb1*+/−); *Apc^min/fl^;Ctnnb1^+/−^* (*min/fl;ctnnb1*+/−); *Apc^min/Δ580^;Ctnnb1^+/−^* (*min/Δ;ctnnb1*+/−). All MEFs were derived from mice on a mixed genetic 129Sv x C57BL/6 background.

To gain biochemical insights into the extent to which Wnt signaling thresholds are related to the tumorigenic response in mice, we generated MEFs of genotypes similar to those of cells having undergone *Apc* LOH in *Apc^min^* mice. In particular, we inactivated the latent *Apc^fl^* allele by Cre-mediated recombination in MEFs following infection with an AdCre-GFP adenovirus that expressed the Cre-recombinase as a GFP-fusion protein (Figure S3). Western blot analysis confirmed expression of the 580 amino acid truncated protein encoded by the recombined *Apc^580Δ^* allele, in the presence of the 850 amino acid Apc^min^ protein (Figure 6A). To prevent our analysis from being affected by potential “plateau effects”, we stimulated MEFs with submaximal concentrations of Wnt3a and found a ∼3-fold increase in Tcf-reporter activity between cells harboring the unrecombined *Apc^fl^* or recombined *Apc^580Δ^* allele, respectively (Figure 6B, compare *Apc^min Δ^ vs. Apc^min/fl^* and *Apc^ min/Δ^;Ctnnb1^+/−^ vs. Apc^min/fl^;Ctnnb1^+/−^*). Furthermore, we confirmed that ablation of one *Ctnnb1* allele reduced reporter activity by approximately 50% (compare *Apc^min/Δ ^vs. Apc^min/Δ^*;*Ctnnb1^+/−^*; *Apc^min/fl^ vs. Apc^min/fl^;Ctnnb1^+/−^* and *Apc ^fl/fl^ vs. Apc^fl/fl^;Ctnnb1^+/−^*), and the comparison suggested similar Tcf4-reponsivenes between *Apc^min/+^* and *Apc^fl/fl^* cells. As predicted from the extent of the activating Apc mutations, we also observed a gradual increase of Tcf reporter activity in the absence of Wnt3a ligand (Figure S4).

**Figure 7:**
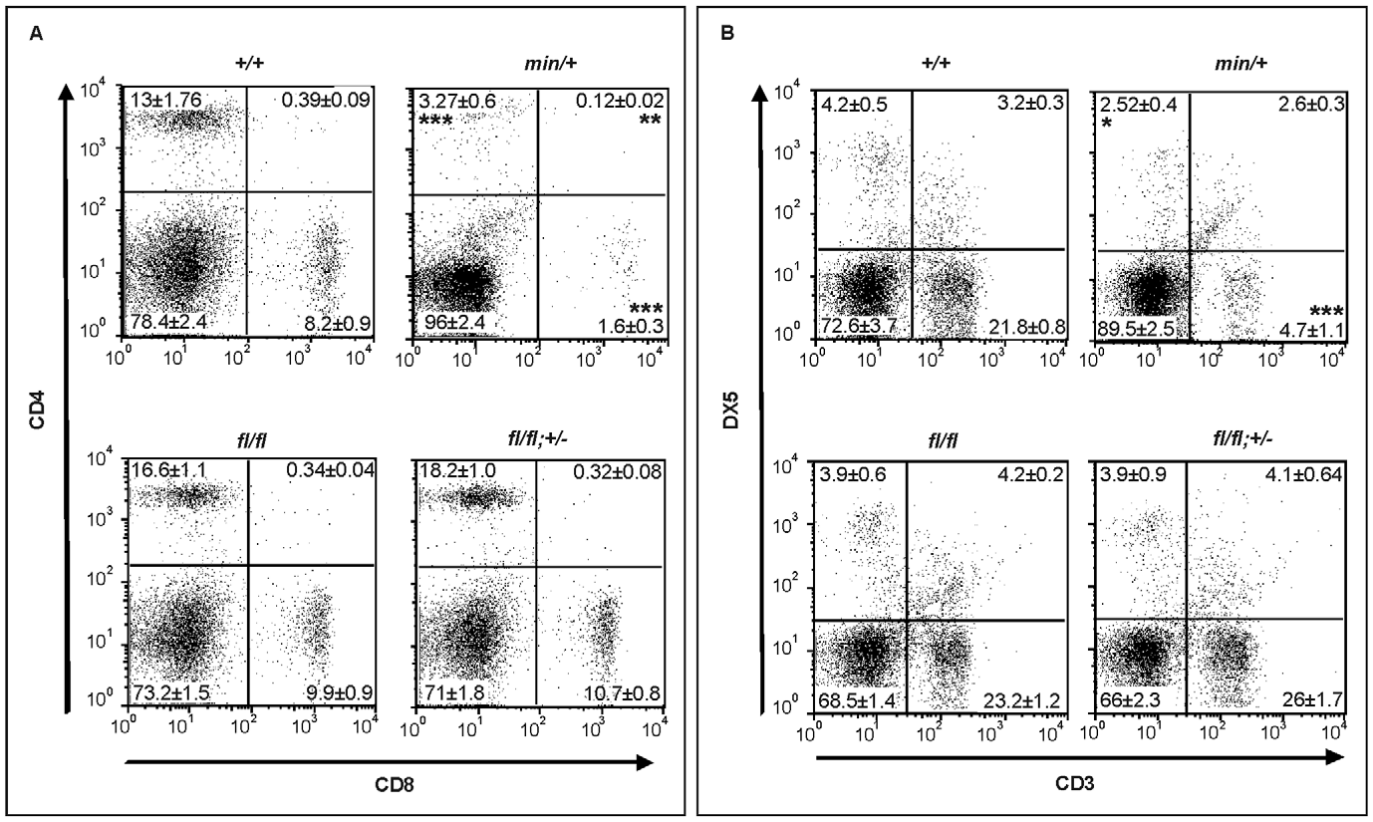
Lymphodepletion in *Apc* hypomorphic mice. (A) Flow cytometry analysis of CD4^+^/CD8^+^ stained splenocytes from 17 week old mice of the indicated genotypes. Representative results from one individual mouse are shown with the percentages contribution to each quadrant shown as Mean ± SD from at least 3 mice. (B) Flow cytometry analysis of CD3^+^/DX5^+^ stained splenocytes from 17 week old mice of the indicated genotypes. The NK cell population (DX5^+^;CD3^−^) are within the top left gate, and the bottom gates contain CD3^+^ T cells. Representative results from one individual mouse are shown with the percentages contribution to each quadrant shown as Mean ± SD from at least 3 mice. Genotypes are as follows: wild-type (*+/+*); *Apc^min/+^* (*min/+*); *Apc^fl/fl^* (*fl/fl*); *Apc^fl/fl^;Ctnnb1^+/−^* (*fl/fl*; +/−). All mice were on a mixed genetic 129Sv x C57BL/6 background.

Since systemic effects observed in adult *Apc^min/+^* mice may arise secondary to LOH-dependent intestinal tumorigenesis, we next used *Apc^fl/fl^* mice to explore this in the context of the Wnt/β-catenin signaling requirement for the maintenance of the hematopoietic cell population [48]. Specifically, *Apc^min/+^* mice develop lymphodepletion around the time when intestinal tumors are observed [49], and this is associated with a progressive loss of immature and mature thymocytes, and the depletion of splenic natural killer (NK) cells. Comparison of 17 week old wild-type, *Apc^fl/fl^;Ctnnb1^+/−^, Apc^fl/fl^* and *Apc^min/+^* mice revealed a strong reduction of mature single positive CD4^+^ and CD8^+^ cells in the spleen of *Apc^min^* mice and a less pronounced reduction in immature double positive CD4^+^,CD8^+^ cells (Figure 7A and Figure S5A). Moreover, this was reflected by a reduction in splenic CD3^+^ thymocytes and DX5^+^, CD3^-^ NK-cells (Figure 7B and Figure S5B) in *Apc^min/+^* mice when compared to *Apc^fl/fl^* mice. Since we did not observe lymphodepletion as a consequence of incremental increases in Wnt/β-catenin signaling from wild-type to *Apc^fl/fl^;Ctnnb1^+/−^* and *Apc^fl/fl^* mice (where the latter allele combination generates comparable signaling to that of *Apc^min/+^* cells), we conclude that this phenotype in aging *Apc^min/+^* mice is likely to be secondary to LOH-induced intestinal tumorigenesis. This conclusion is consistent with the lymphodepletion phenotype persisting in tumor-bearing irradiated *Apc^min/+^* mice that have been reconstituted with wild-type bone marrow [49] and our observation that thymic atrophy and associated T-cell depletion reported by Coletta et al., [49] in their tumor bearing 14 week old *Apc^min/+^* mice is not a reproducible finding at 17 weeks in our *Apc^min/+^* colony (data not shown) which displays a relative delay in polyposis onset.

The present study underscores the power of hypomorphic alleles in the mouse to understand mechanisms that help to explain at the molecular level the specificity of pleiotropic signaling cascades. Here, we propose the existence of differential permissive Wnt/β-catenin signaling threshold levels during development and tissue homeostasis, and how they relate to each other with respect to specific pathophysiological outcomes. Combining biochemical assessment of different *Apc* allele combinations in MEFs with the corresponding mouse phenotype genetically defines threshold levels that are lower for liver tumorigenesis than for influencing cellular identity along the anterior-posterior axis, which in turn are lower than that required for intestinal tumorigenesis (Figure 6B). Our data complement those by Ishikawa et al [50] who observed a more severe head morphogenesis defect in mice homozygous for the hypomorphic *Apc^neoR^* allele which showed an 80% attenuation of full-length Apc protein (compared to ∼70% in *Apc^min/fl^* cells, Figure 1A) and a 7-fold increase in Tcf4-reporter activity (compared to ∼5.5-fold in *Apc^min/fl^* cells, Figure 1B and Figure 6B). Previously, we have shown that functional cooperation between individually insufficient (epi-) genetic alterations induced sufficient aberrant Wnt/β-catenin signaling to trigger intestinal tumorigenesis in compound *A33^Dnmt3a^*;*Apc^min^* mice, with polyps characterized by retention of the wild-type *Apc* allele and epigenetic silencing of the *Sfrp5* gene [51]. Together with the findings presented here, these observations add further support to the “just-right” signaling model which predicts cellular transformation to require specific and distinct dosages of Wnt/β-catenin signaling in intestinal, mammary or hepatic cells, and which was based on the observation that LOH in mice carrying the hypomorphic *Apc^1572T^* allele predisposed to metastatic mammary adenocarcinomas rather than intestinal or hepatic tumorigenesis [52]. Indeed, analysis of somatic mutations found in polyps of FAP patients indicates an active selection process favoring APC genotypes that provide residual levels of β-catenin regulation over its complete loss, which would trigger maximal activation of the Wnt/β-catenin pathway [21]. Furthermore, our results demonstrate a lower requirement of Wnt/β-catenin activation levels for neoplastic transformation of hepatocytes than of intestinal epithelium. Meanwhile, human HCC are frequently associated with somatic mutations in *AXIN1* or *AXIN2* rather than with those in *APC* [36],[37] suggesting that *APC* truncation mutations may be selected against during the process of hepatocyte transformation. Our findings that the frequency of HCC is higher in *Apc^fl/fl^* mice than in *Apc^min/fl^;Ctnnb1^+/−^* mice (despite the higher Tcf reporter activity in MEFs of the latter genotype) may not only be accounted for by the shorter overall survival of *Apc^min/fl^;Ctnnb1^+/−^* mice, but also predicted from the “just-right” signaling model [21],.

Our data also implies that Wnt/β-catenin signaling is likely to conform to cell type-specific bistable switches, where the input stimulus must exceed a threshold to change from one cellular state (and associated response) to another. In the context of *Apc* LOH-dependent intestinal polyposis, for instance, the predicted two-fold increase of Wnt/β-catenin signaling between *Apc^min/Δ^;Ctnnb1^+/−^* cells (corresponding to *Apc^min/LOH^;Ctnnb1^+/−^* lesions in *Apc^min/fl^;Ctnnb1^+/−^* mice) and *Apc^min/Δ^* cells (corresponding to *Apc^min/LOH^* lesions in *Apc^min/+^* mice, Figure 6B), has no further detrimental effect on polyposis-associated survival of *Apc^min/+^* compared to *Apc^min/fl^;Ctnnb1^+/−^* mice (Figure 3D). Indeed, a recent report delineates a nested feedback-loop that may include a Wnt signaling-associated MAPK cascade [53] as one of the components which provides the non-linear input-output relationship for GSK3β and associated Wnt/β-catenin activity [54] to generate the dramatic threshold responses that characterize a bistable system.

Differential sensitivity to genetic dosage provides the basis for establishing therapeutic windows when targeting non-mutated components in diseased tissue. Indeed, for instance, the notion of therapeutic exploitation of non-oncogene addiction is based on the difference in signaling thresholds tolerated between normal and neoplastic cells. Based on our hitherto limited capacity to target and/or compartmentalize drug delivery, global single-allele inactivation models may provide a convenient first screen to identify potential drug targets. Here, we extend this concept from our previous findings for *Stat3* in the context of inflammation-associated gastric cancer [55] to *Ctnnb1* in tumors of the liver and intestine and associated aberrant Wnt signaling.

## Materials and Methods

### Ethics statement

All animals were handled in strict accordance with good animal practice as defined by the relevant national and/or local animal welfare bodies, and all animal work was approved by the appropriate committee.

### Mice

Heterozygous *Ctnnb1^+/−^* mice were generated by excising exons 3–6 from the germline following the mating *Ctnnb1^fl/fl^* males with female C57Bl/6 E2a:Cre mice [56]. *Ryk^+/−^*, the *Apc* mutant *Apc^min/+^* and *Apc^fl/fl^* mice and the BAT-gal transgenic reporter mice have been described previously [22],[23],[57],[58]. All experimental mice were on a mixed genetic 129Sv x C57BL/6 background.

### Quantitative PCR (qPCR) expression analysis

qPCR analysis from liver was performed as described [59]. Following extraction of total RNA with TRIzol reagent (Sigma), first strand complementary DNA was synthesized using the Omniscript RT kit (Qiagen). The PCR reactions were carried out under the following conditions: 94°C for 2 min, denaturation at 92°C for 30 s, annealing at 56°C for 30 s and extension at 72°C for 45 s. Primers were obtained from Invitrogen. The number of cycles was 20 for GAPDH, 25 for Arginase1, Glut2 and RHGB, and 30 for β-catenin and Apc. The calculation of relative expression ratios was carried out with the Relative Expression Software Tool (REST) Multiple Condition Solver (MCS) (http://www.gene-quantification.com/) using the pairwise fixed reallocation randomization test. Primers used are listed in Table S3.

### Tissue fixation, embedding, and processing

Dissected liver tissue was fixed for 1 h in 4% paraformaldehyde or overnight in 10% formalin (Sigma) at 4°C depending on the antibody used (see below). After fixation, tissue samples were transferred to 70% ethanol and embedded in paraffin wax.

### Immunohistochemical analysis of adult

Samples were prepared as described previously [46]. Immunoperoxidase staining for GS, CPS I and CYP2E1 (4% PFA) and β-catenin (formalin) was carried out as follows. Sections were dewaxed in Histoclear for 7 min. Sections were washed in PBS and blocked for 30 min in 2% Roche blocking buffer (Roche) before addition of the following antibodies: anti-mouse GS (1∶400; BD Transduction Laboratories), anti rabbit CPS (1∶1,000; a kind gift of Wouter Lamers), and CYP2E1 (1∶500; a kind gift of Magnus Ingelman-Sundberg) in blocking buffer overnight at 4°C. Immunostaining for β-catenin (1∶50; BD Transduction Laboratories) was carried out as previously described [60]. Excess primary antibody was removed by washing 3 times in PBS for 10 min each. Sections were incubated with the DAKO Envision peroxidase-labeled anti-mouse or rabbit secondary antibody polymer for 30 min. The DAB substrate–chromogen mixture was added to the sections and allowed to develop for 10 min. The reaction was terminated in dH2O and the sections counterstained with hematoxylin where appropriate. Specimens were observed using a Leica DMRB microscope. Image collection from the Leica was made with a Spot camera and images collated into figures in Photoshop.

### Cell culture and transfections

Mouse embryo fibroblasts (MEFs) were derived from E13 embryos and propagated in DMEM supplemented with 10% FBS. The day before transfection, cells were seeded at 5×10^4^ cells/well into 24-well plates. Wnt3a-conditioned medium was a gift from Liz Vincan (Peter MacCallum Cancer Institute, Melbourne) and Nicole Church (JPSL, Ludwig Institute for Cancer Research, Melbourne) and the recombinant human Dkk1- was from R&D Systems (#1090-Dk). Transfections were carried out using either FuGENE 6 transfection reagent (Roche) or nucleofector (Amaxa), 200 ng *pSuperTOPflash*, 4 ng pRL-CMV and 200 ng of pCMV-HA-SFRP5 expression construct. Two days later, cultures were processed using the Dual-Luciferase Reporter Assay kit (Promega) and luminescence was measured using a Lumistar Galaxy luminometer (Dynatech Laboratories).

### Induction of liver carcinogenesis

Mice were injected intraperitoneally with a single dose of diethylnitrosamine (DEN) (10 mg/ml) at 40 mg/kg at 14 days of age. Mice were sacrificed 6–8 months later and livers were scored for the presence of macroscopic tumors.

### Flow cytometry

Single cell suspensions from spleens were prepared by passing organs through a 40 µm mesh. Cell suspensions were treated with NH_4_Cl to lyse red blood cells, and then nonspecific binding was blocked by incubating with mouse Fc block (2.4G2). The cells were incubated for 30 min at RT with the relevant fluorochrome-conjugated antibodies to CD3 (clone 2C11), CD4 (GK1.5), CD8 (53–6.7) and DX5 (#558295). All antibodies and Fc Block for flow cytometry were purchased from BD Biosciences, San Jose, CA. Expression of surface markers on cells was detected using a FACSCalibur flow cytometer (BD Biosciences) and analyzed using the FlowJo software (Tree Star, Inc.) Forward scatter/side scatter (FCS/SSC) gating was used to exclude debris and doublets and dead cells were gated out on the basis of PI positivity measured on the FL-3 channel.

### LacZ staining for embryos

Embryos are killed by submerging in ice-cold PBS for a few minutes and fixed by rocking for 45 min in ice-cold 4% PFA in PBS. Specimens are washed 3×5 min in PBS and subsequently incubated o/n at 30°C in X-gal staining solution. After washing in PBS for a few minutes, stained embryos were photographed.

### Western blotting

Cells were lysed using Triton-X based lysis buffer (30 mM Hepes), 150 mM NaCl, 1% Triton-X-100, 2 mM MgCl_2_), with Complete EDTA-free protease and phosphatase inhibitor cocktail (Roche). This was followed by centrifugation at 13000 g for 5 min at 4°C and denaturing at 95°C for 5 min. Protein concentration was determined using a BIO-RAD assay kit. Proteins were then separated by SDS-PAGE (Invitrogen), blotted onto nitrocellulose and incubated with the appropriate antibody overnight. After incubation with the secondary antibody, proteins were visualized using ECL chemiluminescence detection kit (GE Healthcare). For detection of APC, cell lysates were prepared by resuspending cells in ice-cold Lysis buffer [20 mM HEPES, pH 7.4, 150 mM NaCl, 5 mM EDTA, 1% TritonX-100, 1% deoxycholate and Complete EDTA-free protease inhibitor cocktail] and incubation on ice for 15 min. Lysates were clarified by microcentrifugation at 16,060 *g* for 30 min at 4°C. Total cell lysates were then analysed by SDS-PAGE (3–8% NuPAGE) and detected using the Odyssey infrared imaging system (Odyssey). Quantification of Western blots was performed by using Image J pixel analysis (NIH Image software). Data from Western blots is presented as band density normalized to the loading control, and is representative of three independent experiments. Anti-Active-β-Catenin (anti-ABC), clone 8E7, was from Upstate (#05-665), rabbit polyclonal antibody to the N-terminus of APC (H-290) was obtained from Santa Cruz Biotechnology (Santa Cruz) and anti-mouse Actin (AC-40) was from Sigma-Aldrich.

### Apc LOH determination, promoter methylation analysis

Parts of exon 16 containing the *Min* allele specific T>A substitution was PCR-amplified and the gel-purified amplicons were sequenced on an ABIprism377 DNA sequencer (Applied Biosystems). Apc (ex16) forward primer 5′-TCACCGGAGTAAGCAGAGACAC-3′, reverse primer 5′-TTTGGCATAAGGCATAGAGCAT-3′. Bisulfite treatment of genomic DNA and methylation specific PCR was carried out as described [61].

### Production of adenoviruses and adenoviral infection

Adenovirus expressing Cre Recombinase fused to enhanced green fluorescent protein (GFP; Cre-GFP) was produced by cloning a cDNA encoding Cre-GFP into pShuttle, the adenoviral transfer vector (Q-BIOgene). Linearised plasmid was then co-transformed into *Escherichia coli* with pAdEasy1 (Ad5ΔE1/ΔE3) (Q-BIOgene). The pAdCreGFP was linearised and transfected into Q-HEK293A cells (Q-BIOgene) using the calcium phosphate method (Promega). 10 days after transfection, adenoviral infected cells were collected and the adenovirus was released by three rounds of freeze/thawing, and amplification in Q-HEK293A cells, as described in the protocol (Q-BIOgene). For Tcf4 reporter assays MEFs were plated at 5×10^4^ cells/well and were transfected with p*SuperTOPflash*, and pRenilla-luc. After 24 h, cells were infected with either Ad-LacZ (control virus) or Ad-CreGFP (20 µl/well, TCID50 1.995×10^8^/ml). 48 h after infection, cells were lysed and assayed for luciferase activity. For Western blot analysis, MEFs were plated at 1.5×10^5^ cells/well in 6 well plates and infected with AdCreGFP (20 and 50 µl/well, TCID50 1.995×10^8^/ml) or Ad-LacZ for 48 h. For microscopy, MEFs were plated on glass coverslips, infected with virus, and after 48 h, infected cells were washed twice with PBS and fixed in 4% formaldehyde/PBS for 5 min. DIC and fluorescent images were produced using a Nikon 90i microscope.

### Statistical analysis

Statistical significance was determined by unpaired t-test or, where indicated, using Mann-Whitney analysis.

## Supporting Information

Figure S1 Whole mounts of a representative E13.5 litter derived from mating *Apc^fl/fl^;Ctnnb1^+/+^* with *Apc^min/+^;Ctnnb1^+/−^* mice. N = total number of embryos recovered for the indicated genotypes. Genotypes are as follows: *Apc^+/fl^* (*+/fl*); *Apc^min/fl^* (*min/fl*); *Apc^min/fl^;Ctnnb1^+/−^* (*min/fl;ctnnb1+/−*); *Apc^+/fl^;Ctnnb1^+/−^* (*+/fl;ctnnb1+/−*).
(2.48 MB TIF)

Figure S2 No spontaneous recombination in the liver of *Apc^fl/fl^*; mice in the absence of Cre recombinase. DNA agarose gel of PCR products amplified from DNA derived from normal liver, hepatic tumors or tails from *Apc^fl/fl^* mice on either a *Cre*-deficient (*Cre^−^*) or *Cre*-proficient (*Cre^+^*) background. The 314 bp and the 250 bp products are indicative of unrecombined and loxP-recombined *Apc^fl^* alleles, respectively. L, DNA size ladder; Nrec, non recombined; rec, recombined
(0.73 MB TIF)

Figure S3 Fluorescence analysis of *Apc^min/fl^* MEFs following infection with AdCre-GFP reveals wide-spread nuclear expression of the Cre-GFP fusion protein.
(2.28 MB TIF)

Figure S4 Relative Tcf4 reporter activation in the absence of Wnt3a ligand. Relative Tcf4 reporter activation in MEFs of the indicated genotypes in the absence of Wnt3a ligand. At least two independent experiments were performed in triplicates for each genotype. Mean ± SD. Genotypes are as follows: wild-type (*+/+*); *Apc^fl/fl^;Ctnnb1^+/−^* (*fl/fl*;*ctnnb1+/−*); *Apc^fl/fl^* (*fl/fl*); *Apc^min/+^* (*min*/+); *Apc^min/fl^* (*min/fl*); *Apc^min/Δ580^* (*min/Δ*); *Apc^min/fl^;Ctnnb1^+/−^* (*min/fl;ctnnb1+/−*); *Apc^min/Δ580^;Ctnnb1^+/−^* (*min/Δ;ctnnb1+/−*). All MEFs were derived from mice on a mixed genetic 129Sv x C57BL/6 background.
(3.62 MB TIF)

Figure S5 No lymphodepletion in *Apc* hypomorphic mice. The percentage of single positive CD4^+^, CD8^+^ cells as well as CD4^+^;CD8^+^ double positive splenocytes (A) and CD3^+^ cells and DX5^+^ natural killer cells (B) in mice of the indicated genotypes. Shown are Mean ± SD, n = 3 per genotype, * p<0.05, ** p<0.005, and *** p<0.0001. Genotypes are as follows: wild-type (+/+); *Apc^fl/fl^*;*Ctnnb1* ^+/−^ (*fl/fl*;+/−); *Apc^fl/fl^* (*fl/fl*); *Apc* ^min/+^ (*min*/+). All cells were derived from mice on a mixed genetic 129Sv x C57BL/6 background.
(2.20 MB TIF)

Table S1 Listing of analyzed mouse matings. Number of live embryos (E7.5–E17.5) (A–C) and pups at weaning age (P21) (D) from matings as indicated.
(0.06 MB RTF)

Table S2 Mutational analysis of *Ctnnb1* and *H-Ras* using DNA sequencing. Representative DNA sequencing trails covering *Ctnnb1* exon3 (A) and *H-Ras* (B) of DNA isolated from hepatic tumor lesions (T) or adjacent normal liver tissue (N) of *Apc^fl/fl^* mice. The negative regulatory phosphorylation sites Ser (33, 37, 45) and Thr (41) in β-catenin and the oncogenic hot spot in *H-Ras* affecting codons 12,13 and 61 are indicated in bold.
(0.05 MB RTF)

Table S3 List of primers used for quantitative PCR analysis and Apc LOH determination.
(0.06 MB RTF)
