# Supplementary figures and images for: Genetic Dissection of Differential Signaling Threshold Requirements for the Wnt/β-Catenin Pathway In Vivo
Source: PLoS Genet. 2010 Jan 15;6(1):e1000816. doi: 10.1371/journal.pgen.1000816 (PMC2800045; doi:10.1371/journal.pgen.1000816)

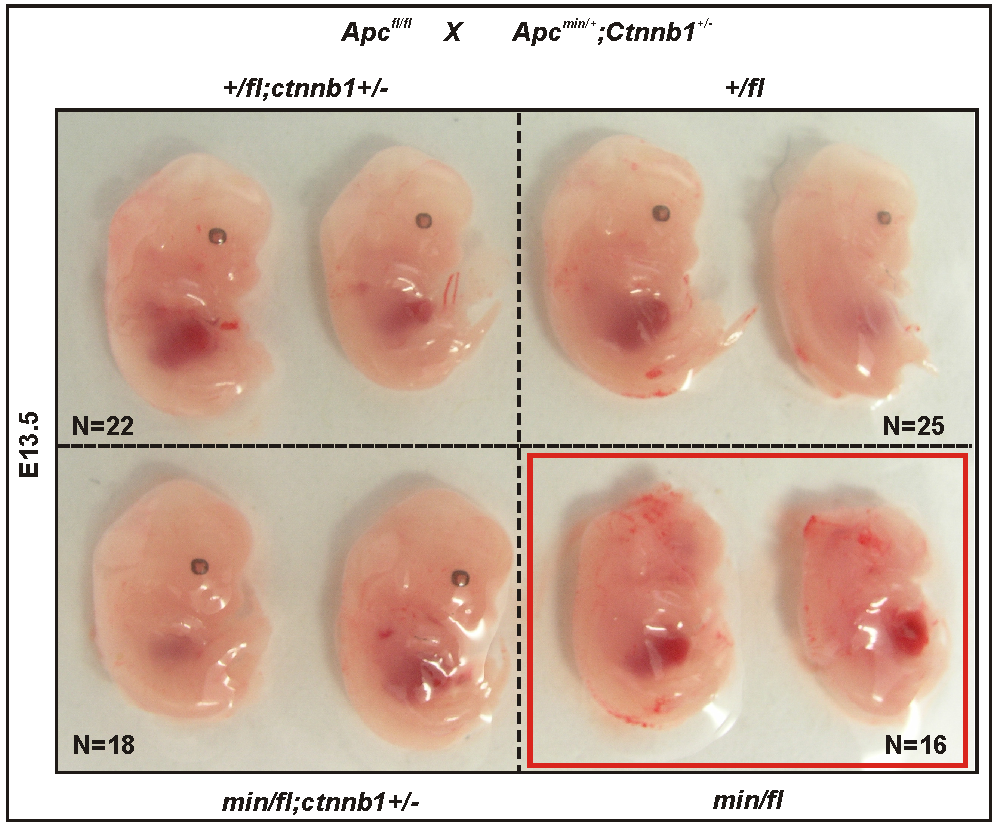

Supplement: Figure S1 — Whole mounts of a representative E13.5 litter derived from mating Apcfl/fl;Ctnnb1+/+ with Apcmin/+;Ctnnb1+/− mice. N = total number of embryos recovered for the indicated genotypes. Genotypes are as follows: Apc+/fl (+/fl); Apcmin/fl (min/fl); Apcmin/fl;Ctnnb1+/− (min/fl;ctnnb1+/−); Apc+/fl;Ctnnb1+/− (+/fl;ctnnb1+/−). (2.48 MB TIF) [file pgen.1000816.s001.tif]

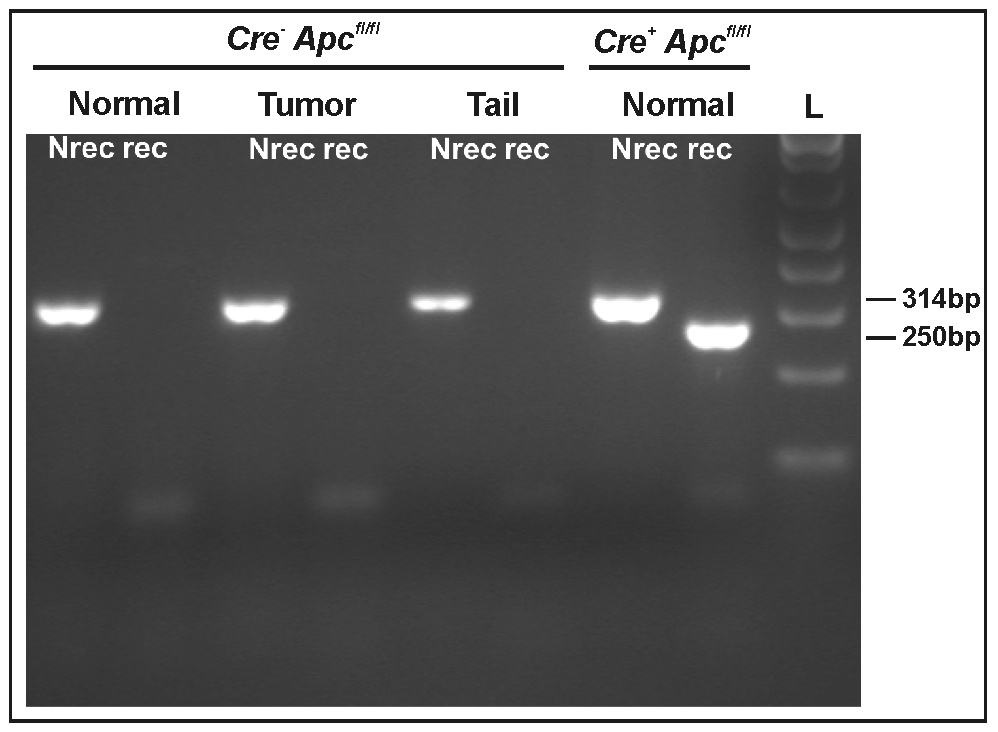

Supplement: Figure S2 — No spontaneous recombination in the liver of Apcfl/fl; mice in the absence of Cre recombinase. DNA agarose gel of PCR products amplified from DNA derived from normal liver, hepatic tumors or tails from Apcfl/fl mice on either a Cre-deficient (Cre−) or Cre-proficient (Cre+) background. The 314 bp and the 250 bp products are indicative of unrecombined and loxP-recombined Apcfl alleles, respectively. L, DNA size ladder; Nrec, non recombined; rec, recombined (0.73 MB TIF) [file pgen.1000816.s002.tif]

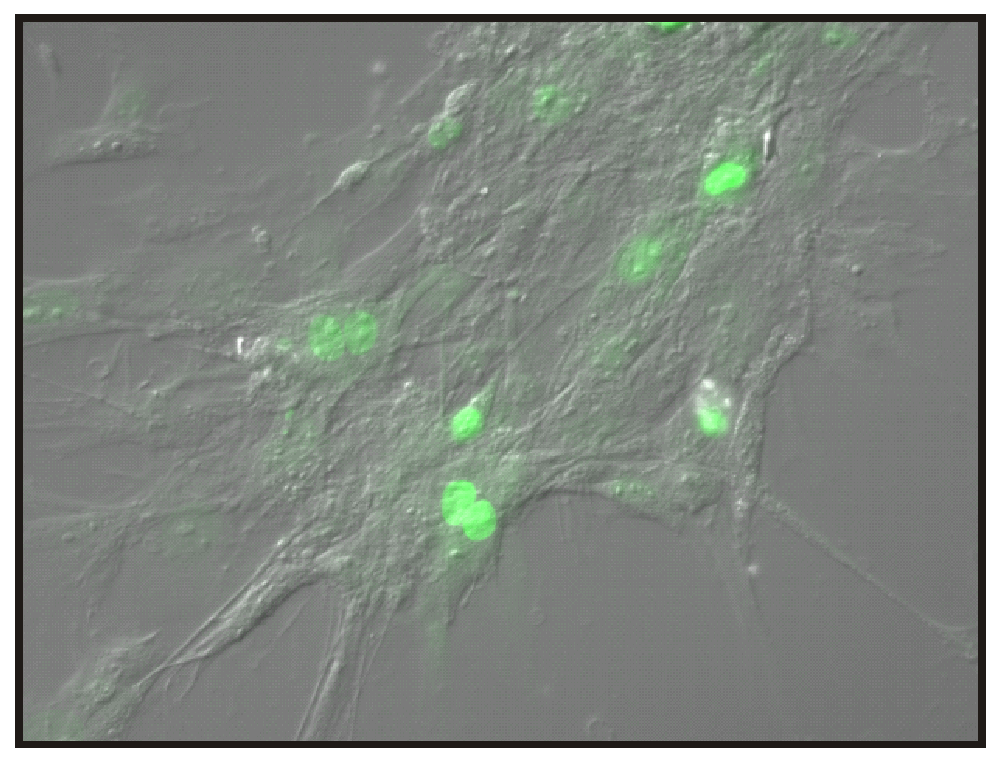

Supplement: Figure S3 — Fluorescence analysis of Apcmin/fl MEFs following infection with AdCre-GFP reveals wide-spread nuclear expression of the Cre-GFP fusion protein. (2.28 MB TIF) [file pgen.1000816.s003.tif]

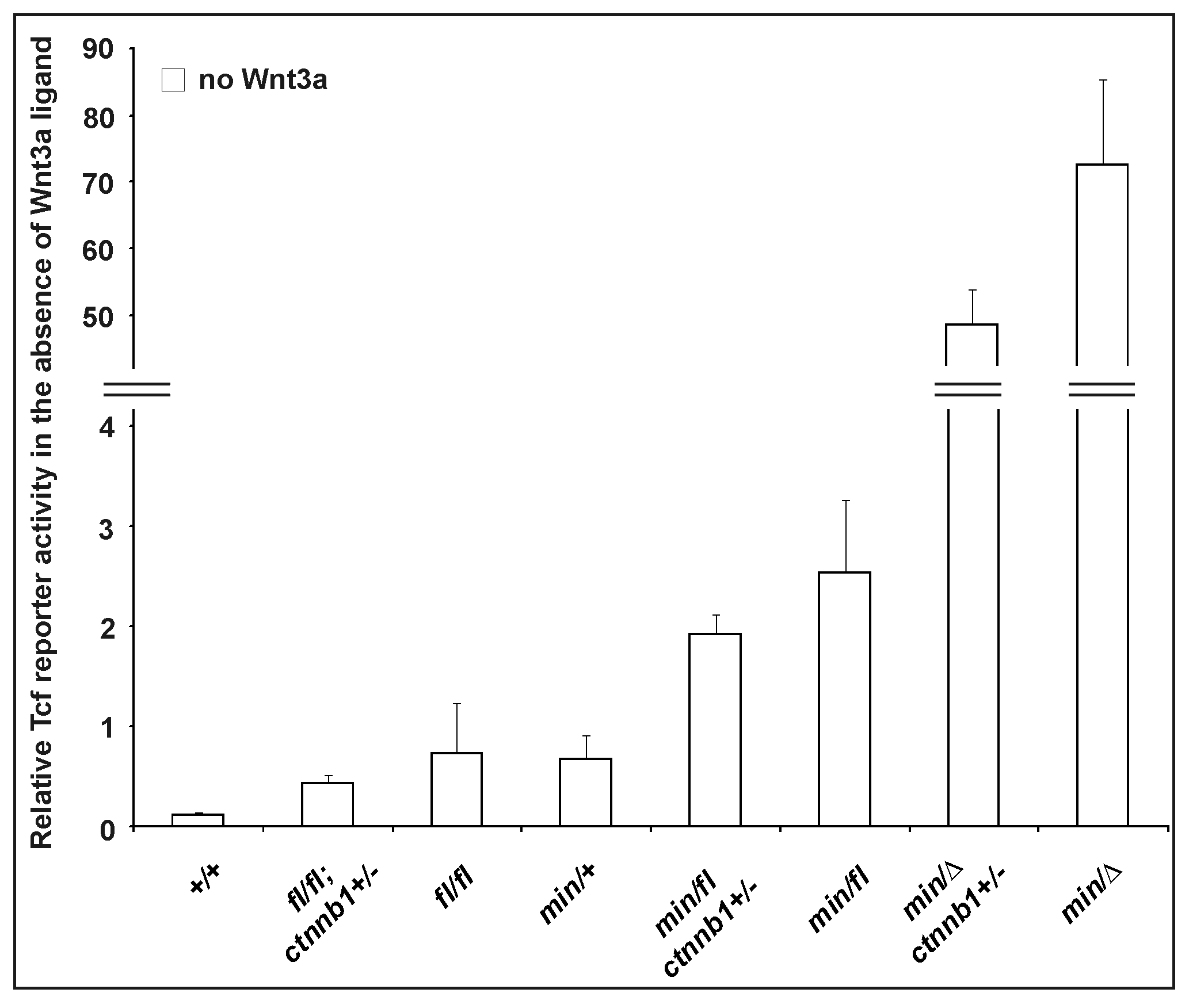

Supplement: Figure S4 — Relative Tcf4 reporter activation in the absence of Wnt3a ligand. Relative Tcf4 reporter activation in MEFs of the indicated genotypes in the absence of Wnt3a ligand. At least two independent experiments were performed in triplicates for each genotype. Mean ± SD. Genotypes are as follows: wild-type (+/+); Apcfl/fl;Ctnnb1+/− (fl/fl;ctnnb1+/−); Apcfl/fl (fl/fl); Apcmin/+ (min/+); Apcmin/fl (min/fl); Apcmin/Δ580 (min/Δ); Apcmin/fl;Ctnnb1+/− (min/fl;ctnnb1+/−); Apcmin/Δ580;Ctnnb1+/− (min/Δ;ctnnb1+/−). All MEFs were derived from mice on a mixed genetic 129Sv x C57BL/6 background. (3.62 MB TIF) [file pgen.1000816.s004.tif]

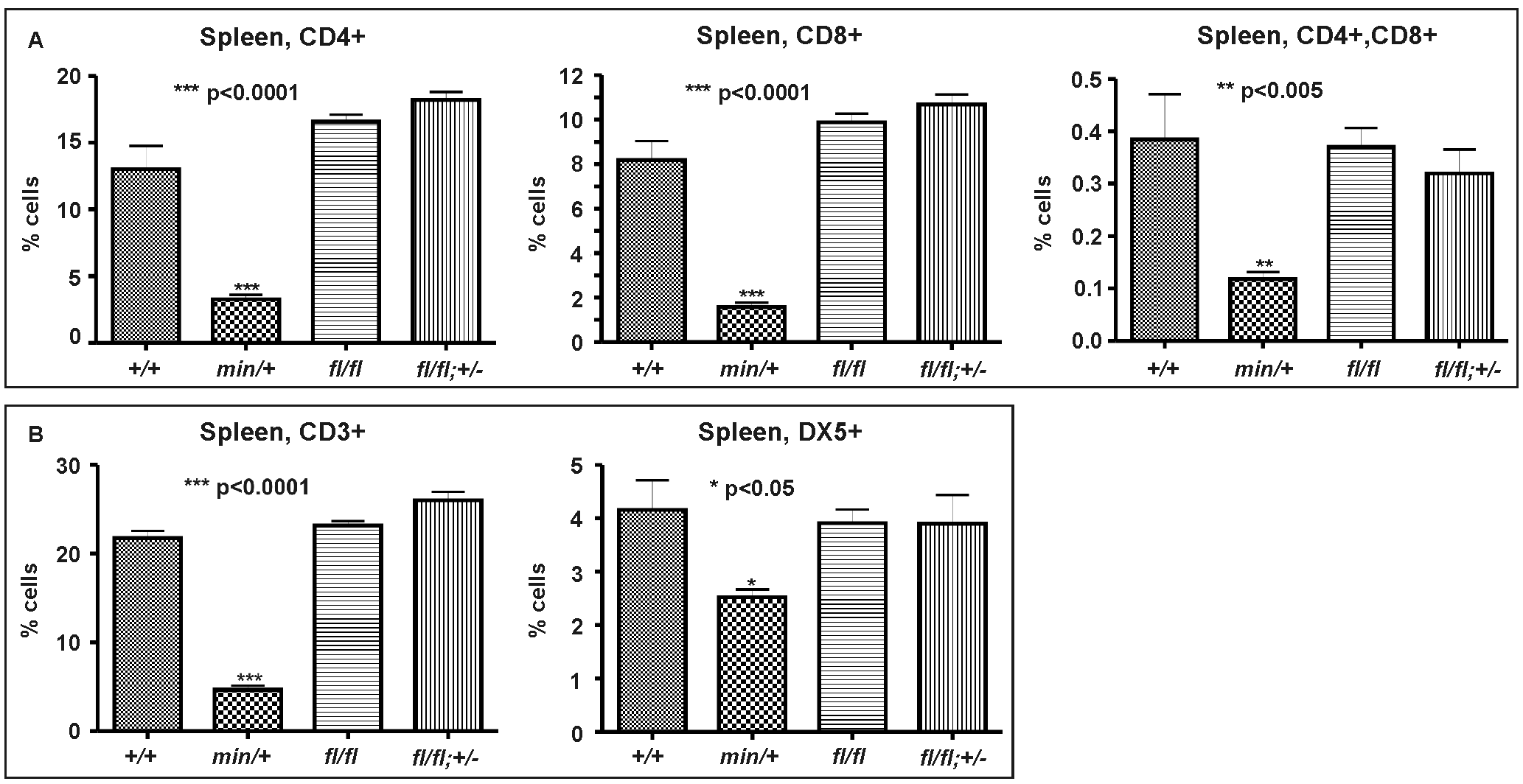

Supplement: Figure S5 — No lymphodepletion in Apc hypomorphic mice. The percentage of single positive CD4+, CD8+ cells as well as CD4+;CD8+ double positive splenocytes (A) and CD3+ cells and DX5+ natural killer cells (B) in mice of the indicated genotypes. Shown are Mean ± SD, n = 3 per genotype, * p<0.05, ** p<0.005, and *** p<0.0001. Genotypes are as follows: wild-type (+/+); Apcfl/fl;Ctnnb1 +/− (fl/fl;+/−); Apcfl/fl (fl/fl); Apc min/+ (min/+). All cells were derived from mice on a mixed genetic 129Sv x C57BL/6 background. (2.20 MB TIF) [file pgen.1000816.s005.tif]
