# Supplementary material for: Genetic Dissection of Differential Signaling Threshold Requirements for the Wnt/β-Catenin Pathway In Vivo
Source: PLoS Genet. 2010 Jan 15;6(1):e1000816. doi: 10.1371/journal.pgen.1000816 (PMC2800045; doi:10.1371/journal.pgen.1000816)
Supplement: Table S1 — Listing of analyzed mouse matings. Number of live embryos (E7.5–E17.5) (A–C) and pups at weaning age (P21) (D) from matings as indicated. (0.06 MB RTF) [file pgen.1000816.s006.rtf]

Table S1

A	Parent 1	Parent 2	
	Apcfl/fl	Apcmin/+	
E7.5 (n=42)	Apc+/fl (n=18)	Apcmin/fl (n=24)	
E9.5 (n=83)	Apc+/fl (n=41)	Apcmin/fl (n=42)	
E13.5 (n=159)	Apc+/fl (n=87)	Apcmin/fl (n=72)	
E15.5 (n=26)	Apc+/fl (n=16)	Apcmin/fl (n=10)	
E17.5 (n=29)	Apc+/fl (n=22)	Apcmin/fl (n=7)	

B	Parent 1	Parent2	
	Apc+/580D	Apc+/fl	
E15.5 (n=30)	Apc+/+ (n=8)	Apc+/580D (n=7)	Apc+/fl (n=10)	Apc580D/fl (n=5)	
P21 (n=66)	Apc+/+ (n=20)	Apc+/580D (n=28)	Apc+/fl (n=18)	Apc580D/fl (n=0)	

C	Parent 1	Parent 2	
	Apcfl/fl;ctnnb1+/-	Apcmin/+;ctnnb1+/+	
E15.5 (n=71)	Apc+/fl;ctnnb1+/+ (n=18)	Apc+/fl;ctnnb1+/- (n=17)	Apcmin/fl;ctnnb1+/+ (n=19)	Apcmin/fl;ctnnb1+/- (n=17)	
P21 (n=85)	Apc+/fl;ctnnb1+/+ (n=28)	Apc+/fl;ctnnb1+/- (n=27)	Apcmin/fl;ctnnb1+/+ (n=0)	Apcmin/fl;ctnnb1+/- (n=30)	

D	Parent 1	Parent 2	
	Apcfl/fl;Ryk+/-	Apcmin/+;Ryk+/-	
P21 (n=55)	Apc+/fl;Ryk+/+ (n=28) Apcmin/fl;Ryk+/+ (n=0)	Apc+/fl;Ryk+/- (n=27) Apcmin/fl;Ryk+/- (n=0)	Apc+/fl;Ryk-/- (n=0) Apcmin/fl;Ryk-/- (n=0)	
