# Supplementary material for: Genetic Dissection of Differential Signaling Threshold Requirements for the Wnt/β-Catenin Pathway In Vivo
Source: PLoS Genet. 2010 Jan 15;6(1):e1000816. doi: 10.1371/journal.pgen.1000816 (PMC2800045; doi:10.1371/journal.pgen.1000816)
Supplement: Table S2 — Mutational analysis of Ctnnb1 and H-Ras using DNA sequencing. Representative DNA sequencing trails covering Ctnnb1 exon3 (A) and H-Ras (B) of DNA isolated from hepatic tumor lesions (T) or adjacent normal liver tissue (N) of Apcfl/fl mice. The negative regulatory phosphorylation sites Ser (33, 37, 45) and Thr (41) in β-catenin and the oncogenic hot spot in H-Ras affecting codons 12,13 and 61 are indicated in bold. (0.05 MB RTF) [file pgen.1000816.s007.rtf]

Table S2

A
AA seq	Asp Ser Gly Ile His Ser Gly Ala Thr Thr Thr Ala Pro Ser	
-catenin exon3	gat tct gga atc cat tct ggt gcc acc acc aca gct cct tcc	
fl/fl Liver N1	GAT TCT GGA ATC CAT TCT GGT GCC ACC ACC ACA GCT CCT TCC	
fl/fl Liver N2	GAT TCT GGA ATC CAT TCT GGT GCC ACC ACC ACA GCT CCT TCC	
fl/fl Liver T1	GAT TCT GGA ATC CAT TCT GGT GCC ACC ACC ACA GCT CCT TCC	
fl/fl Liver T2	GAT TCT GGA ATC CAT TCT GGT GCC ACC ACC ACA GCT CCT TCC	
fl/fl Liver T3	GAT TCT GGA ATC CAT TCT GGT GCC ACC ACC ACA GCT CCT TCC	
fl/fl Liver T4	GAT TCT GGA ATC CAT TCT GGT GCC ACC ACC ACA GCT CCT TCC	
fl/fl Liver T5	GAT TCT GGA ATC CAT TCT GGT GCC ACC ACC ACA GCT CCT TCC	

B
Codon	10  11  12  13  14  15…    //     …59  60  61  62  63	
mu_H-ras	ggc gct gga ggc gtg gga…   //     …gca ggt caa gaa gag	
fl/fl Liver N1	GGC GCT GGA GGC GTG GGA    //      GCA GGT CAA GAA GAG	
fl/fl Liver N2	GGC GCT GGA GGC GTG GGA    //      GCA GGT CAA GAA GAG	
fl/fl Liver T1	GGC GCT GGA GGC GTG GGA    //      GCA GGT CAA GAA GAG	
fl/fl Liver T2	GGC GCT GGA GGC GTG GGA    //      GCA GGT CAA GAA GAG	
fl/fl Liver T3	GGC GCT GGA GGC GTG GGA    //      GCA GGT CAA GAA GAG	
fl/fl Liver T4	GGC GCT GGA GGC GTG GGA    //      GCA GGT CAA GAA GAG	
fl/fl Liver T5	GGC GCT GGA GGC GTG GGA    //      GCA GGT CAA GAA GAG	
