# Supplementary material for: Genetic Dissection of Differential Signaling Threshold Requirements for the Wnt/β-Catenin Pathway In Vivo
Source: PLoS Genet. 2010 Jan 15;6(1):e1000816. doi: 10.1371/journal.pgen.1000816 (PMC2800045; doi:10.1371/journal.pgen.1000816)
Supplement: Table S3 — List of primers used for quantitative PCR analysis and Apc LOH determination. (0.06 MB RTF) [file pgen.1000816.s008.rtf]

Table S3

Gene 	Primer set (5'-3')	Annealing temp. (oC)	
-actin	AGAGGGAAATCGTGCGTGAC
CAATAGTGATGACCTGGCCGT	60	
Hprt1	AGCTACTGTAATGATCAGTCAACG
AGAGGTCCTTTTCACCAGCA	60	
Axin2	GCTCCAGAAGATCACAAAGAGC
AGCTTTGAGCCTTCAGCATC	62	
Dkk2	GTTGGAAGATACTGCCACAG
GGGTCTCCTTCATGTCCTTT	59	
Wif1	GTGTGAACTCAGCAAATGCC
GTAGGGGTTCACCAGATGTA	59	
Cd44	GCCTCAACTGTGCACTCAAA
GTGTTTCAGGGGTGGTCATC	62	
Myc	TGAAGAAGAGCAAGAAGATGAG
CTGGATAGTCCTTCCTTGTG	58	
Apc	TTGATGGAATGTGCTTTGGAA
CACAAGGCTTCCTGGTCTTT	56	
Apc (ex16)	CACCGGAGTAAGCAGAGACAC
TTTGGCATAAGGCATAGAGCAT	60	
Arginase1	GGTTCTGGGAGGCCTATCTT
TTATGGTTACCCTCCCGTTG	56	
-catenin	GCAACCCTGAGGAAGAAGAT
TTAGCTCCTTCCTGATGGAG	56	
GAPDH	TTGTCAGCAATGCATCCTGCACCA
GTCTCCTGTGACTTCAACAGCAAC	56	
Glut2	See reference [46]	56	
Glt1	See reference [46]	56	
RHBG	See reference [46]	56	
